# Supplementary material for: Loss of carnitine palmitoyltransferase 1a reduces docosahexaenoic acid-containing phospholipids and drives sexually dimorphic liver disease in mice
Source: Mol Metab. 2023 Oct 4;78:101815. doi: 10.1016/j.molmet.2023.101815 (PMC10568566; doi:10.1016/j.molmet.2023.101815)
Supplement: Multimedia component 1 [file mmc1.pdf]

Supplemental Table 1. STK Peptide Substrates.

Row, Col	ID	Sequence	Ser	Thr	SpotConc	UniprotAccession	Description
1,1	pTY3H_64_78	RFIGRRQ(pS)LIEDA RK	[71]	[]	50	P07101	Tyrosine 3-monooxygenase (EC 1.14.16.2) (Tyrosine 3-hydroxylase) (TH).
1,2	ATF2_47_59	VADQTPTPTRFLK	[]	[51, 53, 55]	1000	P15336	Cyclic AMP-dependent transcription factor ATF-2 (Activatingtranscription factor 2) (cAMP response element-binding
1,3	CDN1A_139_151	GRKRRQTSMTDFY	[146]	[145, 148]	1000	P38936	Cyclin-dependent kinase inhibitor 1 (p21) (CDK-interacting protein 1) (Melanoma differentiation-associated protein 6) (MDA-6).
1,4	FIBA_569_581	EFPSRGKSSSYSK	[572, 576, 577, 578, 580]	[]	1000	P02671	Fibrinogen alpha chain precursor [Contains: Fibrinopeptide A].
1,5	IKKB_173_185_C179A	LDQGSLATSFVGT	[177, 181]	[180, 185]	1000	O14920	Inhibitor of nuclear factor kappa-B kinase subunit beta (I-kappa-B-kinase beta) (IkBKB) (IKK-beta) (IKK-B) (EC=2.7.11.10) (I-kappa-B kinase 2) (IKK2) (Nuclear factor NF-kappa-B inhibitor kinase beta) (NFKBIKB).
1,6	LIPS_944_956	GFHPRRSSQGATQ	[950, 951]	[955]	1000	Q05469	Hormone-sensitive lipase (HSL) (EC=3.1.1.79).
1,7	MYBB_513_525	DNTPHTPTPFKNA	[]	[515, 518, 520]	1000	P10244	Myb-related protein B (B-Myb).
1,8	PLEK_106_118	GQKFARKSTRRSI	[113, 117]	[114]	1000	P08567	Pleckstrin (Platelet p47 protein).
1,9	RBL2_632_644	DEICIAGSPLTPR	[639]	[642]	1000	Q08999	Retinoblastoma-like protein 2 (130 kDa retinoblastoma-associatedprotein) (p130) (PRB2) (RBR-2).
1,10	VIGLN_289_301	EEKKKKTTTIAVE	[]	[295, 296, 297]	1000	Q00341	Vigilin (High density lipoprotein-binding protein) (HDL-bindingprotein).
1,11	GRIK2_708_720	FMSSRRQSVLVKS	[710, 711, 715, 720]	[]	200	Q13002	Glutamate receptor, ionotropic kainate 2 precursor (Glutamate receptor6) (GluR-6) (GluR6) (Excitatory amino acid receptor 4) (EAA4).
1,12	RADI_559_569	RDKYKTLRQIR	[]	[564]	200	P35241	Radixin.
2,1	ACM1_421_433	CNKAFRDTFRLLL	[]	[428]	1000	P11229	Muscarinic acetylcholine receptor M1.
2,2	ATM_1972_1984	KRSLAFEEGSQST	[1974, 1981, 1983]	[1984]	1000	Q13315	Serine-protein kinase ATM (EC=2.7.11.1) (Ataxia telangiectasia mutated) (A-T, mutated).
2,3	CDN1B_151_163	IRKRPATDDSSTQ	[160, 161]	[157, 162]	1000	P46527	Cyclin-dependent kinase inhibitor 1B (Cyclin-dependent kinase inhibitor p27) (p27Kip1).
2,4	FOXO3_25_37	QSRPRSCTWPLQR	[26, 30]	[32]	1000	O43524	Forkhead box protein O3 (Forkhead in rhabdomyosarcoma-like 1) (AF6q21protein).
2,5	IKKB_686_698	QLMSQPSTASNSL	[689, 692, 695, 697]	[693]	1000	O14920	Inhibitor of nuclear factor kappa-B kinase subunit beta (I-kappa-B-kinase beta) (IkBKB) (IKK-beta) (IKK-B) (EC=2.7.11.10) (I-kappa-B kinase 2) (IKK2) (Nuclear factor NF-kappa-B inhibitor kinase beta) (NFKBIKB).
2,6	LMNA_192_204	DAENRLQTMKEEL	[]	[199]	1000	P02545	Lamin-A/C (70 kDa lamin) (Renal carcinoma antigen NY-REN-32).
2,7	MYC_51_63	KKFELLPTPPLSP	[62]	[58]	1000	P01106	Myc proto-oncogene protein (c-Myc) (Transcription factor p64).
2,8	PP2AB_297_309	EPHVTRRTPDYFL	[]	[301, 304]	1000	P62714	Serine/threonine-protein phosphatase 2A catalytic subunit beta isoform(EC 3.1.3.16) (PP2A-beta).
2,9	RBL2_655_667	GLGRSITSPTTLY	[659, 662]	[661, 664, 665]	1000	Q08999	Retinoblastoma-like protein 2 (130 kDa retinoblastoma-associatedprotein) (p130) (PRB2) (RBR-2).
2,10	YAP1_121_133	QHVRAHSSPASLQ	[127, 128, 131]	[]	1000	P46937	65 kDa Yes-associated protein (YAP65).
2,11	KAP2_92_104	SRFNRRVSVCAET	[92, 99]	[104]	200	P13861	cAMP-dependent protein kinase type II-alpha regulatory subunit.
2,12	RS6_228_240	IAKRRRLSSLRAS	[235, 236, 240]	[]	200	P62753	40S ribosomal protein S6 (Phosphoprotein NP33).
3,1	ACM1_444_456	KIPKRPGSVHRTP	[451]	[455]	1000	P11229	Muscarinic acetylcholine receptor M1.
3,2	BAD_112_124	RELRRMSDEFVDS	[118, 124]	[]	1000	Q92934	Bcl2 antagonist of cell death (BAD) (Bcl-2-binding component 6) (Bcl-XL/Bcl-2-associated death promoter) (Bcl-2-like 8 protein).
3,3	CENPA_1_14	MGPRRRSRKPEAPR	[7]	[]	1000	P49450	Histone H3-like centromeric protein A (Centromere protein A) (CENP-A)(Centromere autoantigen A).

3,4	FRAP_2443_2455	RTRTDSYSAGQSV	[2448, 2450, 2454]	[2444, 2446]	1000	P42345	FKBP12-rapamycin complex-associated protein (FK506-binding protein 12-rapamycin complex-associated protein 1) (Rapamycin target protein) (RAPT1) (Mammalian target of rapamycin) (mTOR).
3,5	K6PL_766_778	LEHVTRRTLSMDK	[775]	[770, 773]	1000	P17858	6-phosphofructokinase, liver type (EC 2.7.1.11) (Phosphofructokinase1) (Phosphohexokinase) (Phosphofructo-1-kinase isozyme B) (PFK-B).
3,6	LMNB1_16_28	GGPTTPLSPTRLS	[23, 28]	[19, 20, 25]	1000	P20700	Lamin-B1.
3,7	NEK2_172_184	FAKTFVGTPYYMS	[184]	[175, 179]	1000	P51955	Serine/threonine-protein kinase Nek2 (EC 2.7.11.1) (NimA-relatedprotein kinase 2) (NimA-like protein kinase 1) (HSPK 21).
3,8	PPR1A_28_40	QIRRRRPTPATLV	[]	[35, 38]	1000	Q13522	Protein phosphatase 1 regulatory subunit 1A (Protein phosphataseinhibitor 1) (IPP-1) (I-1).
3,9	RBL2_959_971	DRTSRDSSPVMRS	[962, 965, 966, 971]	[961]	1000	Q08999	Retinoblastoma-like protein 2 (130 kDa retinoblastoma-associatedprotein) (p130) (PRB2) (RBR-2).
3,10	ADRB2_338_350	ELLCLRRSSLKAY	[345, 346]	[]	200	P07550	Beta-2 adrenergic receptor (Beta-2 adrenoceptor) (Beta-2adrenoreceptor).
3,11	KCC2G_278_289	VASMMHRQETVE	[280]	[287]	200	Q13555	Calcium/calmodulin-dependent protein kinase type II gamma chain(EC 2.7.11.17) (CaM-kinase II gamma chain) (CaM kinase II subunitgamma) (CaMK-II subunit gamma).
3,12	RYR1_4317_4329	VRRLRRLTAREAA	[]	[4324]	200	P21817	Ryanodine receptor 1 (Skeletal muscle-type ryanodine receptor) (RyR1)(RYR-1) (Skeletal muscle calcium release channel).
4,1	ACM4_456_468	CNATFKKTFRHLL	[]	[459, 463]	1000	P08173	Muscarinic acetylcholine receptor M4.
4,2	BAD_69_81	IRSRHSSYPAGTE	[71, 74, 75]	[80]	1000	Q92934	Bcl2 antagonist of cell death (BAD) (Bcl-2-binding component 6) (Bcl-XL/Bcl-2-associated death promoter) (Bcl-2-like 8 protein).
4,3	COF1_17_29	DMKVRKSSTPEEV	[23, 24]	[25]	1000	P23528	Cofilin-1 (Cofilin, non-muscle isoform) (18 kDa phosphoprotein) (p18).
4,4	FRAP_2475_2487	VPESIHSFIGDGL	[2478, 2481]	[]	1000	P42345	FKBP12-rapamycin complex-associated protein (FK506-binding protein 12-rapamycin complex-associated protein 1) (Rapamycin target protein) (RAPT1) (Mammalian target of rapamycin) (mTOR).
4,5	KAPCG_192_206	VKGRTWTLCGTPEYL	[]	[196, 198, 202]	1000	P22612	cAMP-dependent protein kinase catalytic subunit gamma (EC 2.7.11.11)(PKA C-gamma).
4,6	MARCS_152_164	KKKKKRFSFKKSF	[159, 163]	[]	1000	P29966	Myristoylated alanine-rich C-kinase substrate (MARCKS) (Protein kinaseC substrate, 80 kDa protein, light chain) (PKCSL) (80K-L protein).
4,7	NEK3_158_170	FACTYVGTPYYVP	[]	[161, 165]	1000	P51956	Serine/threonine-protein kinase Nek3 (EC 2.7.11.1) (NimA-relatedprotein kinase 3) (HSPK 36).
4,8	PRKDC_2618_2630	TRTQEGSLSARWP	[2624, 2626]	[2618, 2620]	1000	P78527	DNA-dependent protein kinase catalytic subunit (DNA-PK catalytic subunit) (DNA-PKcs) (EC=2.7.11.1) (DNPK1) (p460).
4,9	REL_260_272	KMQLRRPSDQEVS	[267, 272]	[]	1000	Q04864	C-Rel proto-oncogene protein (C-Rel protein).
4,10	ART_025_CXGLRRWSLGGLRRWSL	GLRRWSLGGLRRWSL	NA	NA	200	NA	NA
4,11	KCNA1_438_450	DSDLSRRSSSTMS	[439, 442, 445, 446, 447, 450]	[448]	200	Q09470	Potassium voltage-gated channel subfamily A member 1 (Voltage-gatedpotassium channel subunit Kv1.1) (HUKI) (HBK1).
4,12	SCN7A_898_910	KNGCRRGSSLGQI	[905, 906]	[]	200	Q01118	Sodium channel protein type 7 subunit alpha (Sodium channel proteintype VII subunit alpha) (Putative voltage-gated sodium channel subunitalpha Nax) (Sodium channel protein cardiac and skeletal muscle
5,1	ACM5_494_506	CYALCNRTFRKTF	[]	[501, 505]	1000	P08912	Muscarinic acetylcholine receptor M5.
5,2	BAD_93_105	FRGRSRSAPPNLW	[97, 99]	[]	1000	Q92934	Bcl2 antagonist of cell death (BAD) (Bcl-2-binding component 6) (Bcl-XL/Bcl-2-associated death promoter) (Bcl-2-like 8 protein).
5,3	CSF1R_701_713	NIHLEKKYVRRDS	[713]	[]	1000	P07333	Macrophage colony-stimulating factor 1 receptor precursor(EC 2.7.10.1) (CSF-1-R) (Fms proto-oncogene) (c-fms) (CD115 antigen).
5,4	GPR6_349_361	QSKVPFRSRSPSE	[350, 356, 358, 360]	[]	1000	P46095	Sphingosine 1-phosphate receptor GPR6 (G-protein coupled receptor 6).
5,5	KCNA2_442_454	PDLKKSRSASTIS	[447, 449, 451, 454]	[452]	1000	P16389	Potassium voltage-gated channel subfamily A member 2 (Voltage-gatedpotassium channel subunit Kv1.2) (HBK5) (NGK1) (HUKIV).

5,6	MARCS_160_172	FKKSFKLSGFSFK	[163, 167, 170]	[]	1000	P29966	Myristoylated alanine-rich C-kinase substrate (MARCKS) (Protein kinaseC substrate, 80 kDa protein, light chain) (PKCSL) (80K-L protein).
5,7	NMDZ1_890_902	SFKRRRSSKDTST	[890, 896, 897, 901]	[900, 902]	1000	Q05586	Glutamate [NMDA] receptor subunit zeta-1 precursor (N-methyl-D-aspartate receptor subunit NR1).
5,8	PTK6_436_448	ALRERLSSFTSYE	[442, 443, 446]	[445]	1000	Q13882	Tyrosine-protein kinase 6 (EC 2.7.10.2) (Breast tumor kinase)(Tyrosine-protein kinase BRK).
5,9	SRC_413_425	LIEDNEYTARQGA	[]	[420]	1000	P12931	Proto-oncogene tyrosine-protein kinase Src (EC 2.7.10.2) (p60-Src) (c-Src) (pp60c-src).
5,10	CAC1C_1974_1986	ASLGRRASFHLEC	[1975, 1981]	[]	200	Q13936	Voltage-dependent L-type calcium channel subunit alpha-1C (Voltage-gated calcium channel subunit alpha Cav1.2) (Calcium channel, L type,alpha-1 polypeptide, isoform 1, cardiac muscle).
5,11	KPB1_1011_1023	QVEFRRLSISAES	[1018, 1020, 1023]	[]	200	P46020	Phosphorylase b kinase regulatory subunit alpha, skeletal muscleisoform (Phosphorylase kinase alpha M subunit).
5,12	SRC8_CHICK_423_435	KTPSSPVYQDAVS	[426, 427, 435]	[424]	200	Q01406	Src substrate protein p85 (p80) (Cortactin).
6,1	ACM5_498_510	CNRTFRKTFKMLL	[]	[501, 505]	1000	P08912	Muscarinic acetylcholine receptor M5.
6,2	BCKD_45_57	ERSKTVTSFYNQS	[47, 52, 57]	[49, 51]	1000	O14874	[3-methyl-2-oxobutanoate dehydrogenase [lipoamide]] kinase,mitochondrial precursor (EC 2.7.11.4) (Branched-chain alpha-ketoaciddehydrogenase kinase) (BCKDHKIN) (BCKD-kinase).
6,3	CSK21_355_367	ISSVPTPSPLGPL	[356, 357, 362]	[360]	1000	P68400	Casein kinase II subunit alpha (EC 2.7.11.1) (CK II).
6,4	GPSM2_394_406	PKLGRRHSMENME	[401]	[]	1000	P81274	G-protein-signaling modulator 2 (Mosaic protein LGN).
6,5	KCNA3_461_473	EELRKARSNSTLS	[468, 470, 473]	[471]	1000	P22001	Potassium voltage-gated channel subfamily A member 3 (Voltage-gatedpotassium channel subunit Kv1.3) (HPCN3) (HGK5) (HuKIII) (HLK3).
6,6	MBP_222_234	HFFKNIVTPRTPP	[]	[229, 232]	1000	P02686	Myelin basic protein (MBP) (Myelin A1 protein) (Myelin membraneencephalitogenic protein).
6,7	NOS3_1171_1183	SRIRTQSFSLQER	[1171, 1177, 1179]	[1175]	1000	P29474	Nitric oxide synthase, endothelial (EC=1.14.13.39) (Endothelial NOS) (eNOS) (EC-NOS) (NOS type III) (NOSIII) (Constitutive NOS) (cNOS).
6,8	RAB1A_187_199	KSNVKIQSTPVKQ	[188, 194]	[195]	1000	P62820	Ras-related protein Rab-1A (YPT1-related protein).
6,9	STK6_283_295	SSRRTTLCGTLDY	[283, 284]	[287, 288, 292]	1000	O14965	Serine/threonine-protein kinase 6 (EC 2.7.11.1) (Aurora kinase A)(Aurora-A) (Serine/threonine kinase 15) (Aurora/IPL1-related kinase 1)(Aurora-related kinase 1) (hARK1) (Breast tumor-amplified kinase).
6,10	CFTR_730_742	EPLERRLSLVPDS	[737, 742]	[]	200	P13569	Cystic fibrosis transmembrane conductance regulator (CFTR) (cAMP-dependent chloride channel) (ATP-binding cassette transporter sub-
6,11	MYPC3_268_280	LSAFRRTSLAGGG	[269, 275]	[274]	200	Q14896	Myosin-binding protein C, cardiac-type (Cardiac MyBP-C) (C-protein,cardiac muscle isoform).
6,12	VASP_271_283	LARRRKATQVGEK	[]	[278]	200	P50552	Vasodilator-stimulated phosphoprotein (VASP).
7,1	ADDB_696_708	GSPSKSPSKKKKK	[697, 699, 701, 703]	[]	1000	P35612	Beta-adducin (Erythrocyte adducin subunit beta)
7,2	BRCA1_1451_1463	EKAVLTSQKSSEY	[1457, 1460, 1461]	[1456]	1000	P38398	Breast cancer type 1 susceptibility protein (RING finger protein 53).
7,3	DCX_49_61	HFDERDKTSRNMR	[57]	[56]	1000	O43602	Neuronal migration protein doublecortin (Lissencephalin-X) (Lis-X)(Doublin).
7,4	GSUB_61_73	KKPRRKDTPALHI	[]	[68]	1000	O96001	G-substrate.
7,5	KCNB1_489_501	KWTKRTLSETSSS	[496, 499, 500, 501]	[491, 494, 498]	1000	Q14721	Potassium voltage-gated channel subfamily B member 1 (Voltage-gatedpotassium channel subunit Kv2.1) (h-DRK1).
7,6	MK10_214_226	AGTSFMMTPYVVT	[217]	[216, 221, 226]	1000	P53779	Mitogen-activated protein kinase 10 (EC 2.7.11.24) (Stress-activated protein kinase JNK3) (c-Jun N-terminal kinase 3) (MAP kinase p49 3F12).
7,7	NR4A1_344_356	GRRGRLPSKPKQP	[351]	[]	1000	P22736	Nuclear receptor subfamily 4 group A member 1 (Orphan nuclear receptorHMR) (Early response protein NAK1) (TR3 orphan receptor) (ST-
7,8	RAF1_253_265	QRQRSTSTPNVHM	[257, 259]	[258, 260]	1000	P04049	RAF proto-oncogene serine/threonine-protein kinase (EC 2.7.11.1) (Raf-1) (C-RAF) (cRaf).

7,9	STMN2_90_102	AAGERRKSQEAQV	[97]	[]	1000	Q93045	Stathmin-2 (Protein SCG10) (Superior cervical ganglion-10 protein).
7,10	CGHB_109_121	QCALCRRSTTDCG	[116]	[117, 118]	200	P01233	Choriogonadotropin subunit beta precursor (CG-beta) (Chorionicgonadotrophin chain beta).
7,11	NCF1_296_308	RGAPPRRSSIRNA	[303, 304]	[]	200	P14598	Neutrophil cytosol factor 1 (NCF-1) (Neutrophil NADPH oxidase factor1) (47 kDa neutrophil oxidase factor) (p47-phox) (NCF-47K) (47 kDaautosomal chronic granulomatous disease protein) (Nox organizer 2)(Nox-organizing protein 2) (SH3 and PX domain-containing protein
7,12	VTNC_390_402	NQNSRRPSRATWL	[393, 397]	[400]	200	P04004	Vitronectin precursor (Serum-spreading factor) (S-protein) (V75)[Contains: Vitronectin V65 subunit; Vitronectin V10
8,1	ADDB_706_718	KKKFRTPSFLKKS	[713, 718]	[711]	1000	P35612	Beta-adducin (Erythrocyte adducin subunit beta)
8,2	C1R_201_213	ASGYISSLEYPRS	[202, 206, 207, 213]	[]	1000	P00736	Complement C1r subcomponent (EC=3.4.21.41)
8,3	ELK1_356_368	LLPTHTLTPVLLT	[]	[359, 361, 363, 368]	1000	P19419	ETS domain-containing protein Elk-1.
8,4	GYS2_1_13	MLRGRSLSVTSLG	[6, 8, 11]	[10]	1000	P54840	Glycogen [starch] synthase, liver (EC 2.4.1.11).
8,5	KIF11_919_931	LDIPTGTTPQRKS	[931]	[923, 925, 926]	1000	P52732	Kinesin-like protein KIF11 (Kinesin-related motor protein Eg5)(Kinesin-like spindle protein HKSP) (Thyroid receptor-interactingprotein 5) (TRIP-5) (Kinesin-like protein 1).
8,6	MP2K1_281_293	GDAAETPPRPRTP	[]	[286, 292]	1000	Q02750	Dual specificity mitogen-activated protein kinase kinase 1(EC 2.7.12.2) (MAP kinase kinase 1) (MAPKK 1) (ERK activator kinase 1)(MAPK/ERK
8,7	NTRK3_824_836	LHALGKATPIYLD	[]	[831]	1000	Q16288	NT-3 growth factor receptor precursor (EC 2.7.10.1) (Neurotrophictyrosine kinase receptor type 3) (TrkC tyrosine kinase)
8,8	RAP1B_172_184	PGKARKKSSCQLL	[179, 180]	[]	1000	P61224	Ras-related protein Rap-1b precursor (GTP-binding protein smg p21B).
8,9	TAU_524_536	GSRSRTPSLPTPP	[525, 527, 531]	[529, 534]	1000	P10636	Microtubule-associated protein tau (Neurofibrillary tangle protein)(Paired helical filament-tau) (PHF-tau).
8,10	CREB1_126_138	EILSRRPSYRKIL	[129, 133]	[]	200	P16220	cAMP response element-binding protein (CREB).
8,11	NCF1_321_333	QDAYRRNSVRFLQ	[328]	[]	200	P14598	Neutrophil cytosol factor 1 (NCF-1) (Neutrophil NADPH oxidase factor1) (47 kDa neutrophil oxidase factor) (p47-phox) (NCF-47K) (47 kDaautosomal chronic granulomatous disease protein) (Nox organizer 2)(Nox-organizing protein 2) (SH3 and PX domain-containing protein
8,12	CFTR_761_773	LQARRRQSVLNLM	[768]	[]	200	P13569	Cystic fibrosis transmembrane conductance regulator (CFTR) (cAMP-dependent chloride channel) (ATP-binding cassette transporter sub-
9,1	AKT1_301_313	KDGATMKTFCGTP	[]	[305, 308, 312]	1000	P31749	RAC-alpha serine/threonine-protein kinase (EC 2.7.11.1) (RAC-PK-alpha)(Protein kinase B) (PKB) (C-AKT).
9,2	CA2D1_494_506	LEDIKRLTPRFTL	[]	[501, 505]	1000	P54289	Voltage-dependent calcium channel subunit alpha-2/delta-1 precursor(Voltage-gated calcium channel subunit alpha-2/delta-1) [Contains:Voltage-dependent calcium channel subunit alpha-2-1;
9,3	ELK1_410_422	ISVDGLSTPVVLS	[411, 416, 422]	[417]	1000	P19419	ETS domain-containing protein Elk-1.
9,4	H2B1B_ 27_40	GKKRKRSRKESYSI	[33, 37, 39]	[]	1000	P33778	Histone H2B type 1-B (H2B.f) (H2B/f) (H2B.1).
9,5	KIF2C_105_118_S106G	EGLRSRSTRMSTVS	[109, 111, 115, 118]	[112, 116]	1000	Q99661	Kinesin-like protein KIF2C (Mitotic centromere-associated kinesin)(MCAK) (Kinesin-like protein 6).
9,6	MP2K1_287_299	PPRPRTPGRPLSS	[298, 299]	[292]	1000	Q02750	Dual specificity mitogen-activated protein kinase kinase 1(EC 2.7.12.2) (MAP kinase kinase 1) (MAPKK 1) (ERK activator kinase 1)(MAPK/ERK
9,7	P53_12_24	PPLSQETFSDLWK	[15, 20]	[18]	1000	P04637	Cellular tumor antigen p53 (Tumor suppressor p53) (Phosphoprotein p53)(Antigen NY-CO-13).
9,8	RB_242_254	AVIPINGSPRTPR	[249]	[252]	1000	P06400	Retinoblastoma-associated protein (PP110) (P105-RB) (RB).
9,9	TLE2_246_258	EPPSPATTPCGKV	[249]	[252, 253]	1000	Q04725	Transducin-like enhancer protein 2 (ESG2).

9,10	DESP_2842_2854	RSGSRRGSFDATG	[2843, 2845, 2849]	[2853]	200	P15924	Desmoplakin (DP) (250/210 kDa paraneoplastic pemphigus antigen).
9,11	NFKB1_330_342	FVQLRRKSDLETS	[337, 342]	[341]	200	P19838	Nuclear factor NF-kappa-B p105 subunit (DNA-binding factor KBF1) (EBP-1) [Contains: Nuclear factor NF-kappa-B p50 subunit].
9,12	F263_454_466	NPLMRRNSVTPLA	[461]	[463]	200	Q16875	6-phosphofructo-2-kinase/fructose-2,6-biphosphatase 3 (6PF-2-K/Fru-2,6-P2ASE brain/placenta-type isozyme) (iPFK-2) (Renal carcinomaantigen NY-REN-56) [Includes: 6-phosphofructo-2-kinase (EC 2.7.1.105);Fructose-2,6-bisphosphatase (EC 3.1.3.46)].
10,1	ANDR_785_797	VRMRHLSQEFGWL	[791]	[]	1000	P10275	Androgen receptor (Dihydrotestosterone receptor) (Nuclear receptor subfamily 3 group C member 4).
10,2	CD27_212_224	HQRRKYRSNKGES	[219, 224]	[]	1000	P26842	CD27 antigen precursor (CD27L receptor) (T-cell activation antigenCD27) (T14) (Tumor necrosis factor receptor superfamily
10,3	ERBB2_679_691	QQKIRKYTMRRLL	[]	[686]	1000	P04626	Receptor tyrosine-protein kinase erbB-2 precursor (EC 2.7.10.1)(p185erbB2) (C-erbB-2) (NEU proto-oncogene) (Tyrosine kinase-type cellsurface receptor HER2) (MLN 19) (CD340 antigen).
10,4	H32_3_18	RTKQTARKSTGGKAPR	[11]	[4, 7, 12]	1000	Q71DI3	Histone H3.2 (H3/m) (H3/o).
10,5	KPCB_19_31_A25S	RFARKGSLRQKNV	[25]	[]	1000	P05771	Protein kinase C beta type (EC 2.7.11.13) (PKC-beta) (PKC-B).
10,6	MPH6_140_152	EDENGDITPIKAK	[]	[147]	1000	Q99547	M-phase phosphoprotein 6.
10,7	P53_308_323	LPNNTSSSPQPKKKPL	[313, 314, 315]	[312]	1000	P04637	Cellular tumor antigen p53 (Tumor suppressor p53) (Phosphoprotein p53)(Antigen NY-CO-13).
10,8	RB_350_362	SFETQRTPRKSNL	[350, 360]	[353, 356]	1000	P06400	Retinoblastoma-associated protein (PP110) (P105-RB) (RB).
10,9	TOP2A_1463_1475	RRKRKPSTSDDSD	[1469, 1471, 1474]	[1470]	1000	P11388	DNA topoisomerase 2-alpha (EC=5.99.1.3) (DNA topoisomerase II, alpha isozyme).
10,10	E1A_ADE05_212_224	AILRRPTSPVSRE	[219, 222]	[218]	200	P03255	Early E1A 32 kDa protein.
10,11	PLM_76_88	EEGTFRSSIRRLS	[82, 83, 88]	[79]	200	O00168	Phospholemman precursor (FXYD domain-containing ion transportregulator 1).
10,12	KAP3_107_119	NRFTRRASVCAEA	[114]	[110]	200	P31323	cAMP-dependent protein kinase type II-beta regulatory subunit.
11,1	ANXA1_209_221	AGERRKGTDVNVF	[]	[216]	1000	P04083	Annexin A1 (Annexin-1) (Annexin I) (Lipocortin I) (Calpactin II)(Chromobindin-9) (p35) (Phospholipase A2 inhibitory protein).
11,2	CDC2_154_169	GIPIRVYTHEVVTLWY	[]	[161, 166]	1000	P06493	Cell division control protein 2 homolog (EC 2.7.11.22) (EC 2.7.11.23) (p34 protein kinase) (Cyclin-dependent kinase 1) (CDK1).
11,3	ERF_519_531	GEAGGPLTPRRVS	[531]	[526]	1000	P50548	ETS domain-containing transcription factor ERF (Ets2 repressorfactor).
11,4	IF4E_203_215	TATKSGSTTKNRF	[207, 209]	[203, 205, 210, 211]	1000	P06730	Eukaryotic translation initiation factor 4E (eIF-4E) (eIF4E) (mRNA cap-binding protein) (eIF-4F 25 kDa subunit).
11,5	KPCB_626_639	AENFDRFFTRHPPV	[]	[634]	1000	P05771-2	Protein kinase C beta type (EC 2.7.11.13) (PKC-beta) (PKC-B).
11,6	MPIP1_172_184	FTQRQNSAPARML	[178]	[173]	1000	P30304	M-phase inducer phosphatase 1 (EC=3.1.3.48) (Dual specificity phosphatase Cdc25A).
11,7	PDE5A_95_107	GTPTRKISASEFD	[102, 104]	[96, 98]	1000	O76074	cGMP-specific 3',5'-cyclic phosphodiesterase (EC 3.1.4.35) (CGB-PDE)(cGMP-binding cGMP-specific phosphodiesterase).
11,8	RB_774_786	TRPPTLSPIPHIP	[780]	[774, 778]	1000	P06400	Retinoblastoma-associated protein (PP110) (P105-RB) (RB).
11,9	VASP_150_162	EHIERRVSNAGGP	[157]	[]	1000	P50552	Vasodilator-stimulated phosphoprotein (VASP).
11,10	EPB42_241_253	LLNKRRGSVPILR	[248]	[]	200	P16452	Erythrocyte membrane protein band 4.2 (Erythrocyte protein 4.2)(P4.2).
11,11	PTN12_32_44	FMRLRRLSTKYRT	[39]	[40, 44]	200	Q05209	Tyrosine-protein phosphatase non-receptor type 12 (EC 3.1.3.48)(Protein-tyrosine phosphatase G1) (PTPG1) (PTP-PEST).
11,12	KCNA6_504_516	ANRERRPSYLPTP	[511]	[515]	200	P17658	Potassium voltage-gated channel subfamily A member 6 (Voltage-gatedpotassium channel subunit Kv1.6) (HBK2).

12,1	pVASP_150_164	EHIERRV(pS)NAGG PPA	[157]	[]	50	P50552	Vasodilator-stimulated phosphoprotein (VASP).
12,2	CDK7_163_175	GSPNRAYTHQVVT	[164]	[170, 175]	1000	P50613	Cell division protein kinase 7 (EC 2.7.11.22) (EC 2.7.11.23) (CDK-activating kinase) (CAK) (TFIIH basal transcription factor complexkinase subunit) (39 kDa protein kinase) (P39 Mo15) (STK1) (CAK1).
12,3	ESR1_160_172	GGRERLASTNDKG	[167]	[168]	1000	P03372	Estrogen receptor (ER) (Estradiol receptor) (ER-alpha) (Nuclearreceptor subfamily 3 group A member 1).
12,4	IKBA_26_38	LDDRHDSGLDSMK	[32, 36]	[]	1000	P25963	NF-kappa-B inhibitor alpha (I-kappa-B-alpha) (IkappaBalpha) (IkB-alpha) (Major histocompatibility complex enhancer-binding protein MAD3).
12,5	KS6A1_374_386	QLFRGFSFVATGL	[380]	[384]	1000	Q15418	Ribosomal protein S6 kinase alpha-1 (S6K-alpha 1) (EC=2.7.11.1) (90 kDa ribosomal protein S6 kinase 1) (p90-RSK 1) (pp90RSK1) (p90S6K) (Ribosomal S6 kinase 1) (RSK-1) (MAP kinase-activated protein kinase
12,6	MPIP3_208_220	RSGLYRSPSMPEN	[209, 214, 216]	[]	1000	P30307	M-phase inducer phosphatase 3 (EC 3.1.3.48) (Dual specificityphosphatase Cdc25C).
12,7	PDPK1_27_39	SMVRTQTESSTPP	[27, 35, 36]	[31, 33, 37]	1000	O15530	3-phosphoinositide-dependent protein kinase 1 (EC 2.7.11.1) (hPDK1).
12,8	RB_803_815	NIYISPLKSPYKI	[807, 811]	[]	1000	P06400	Retinoblastoma-associated protein (PP110) (P105-RB) (RB).
12,9	VASP_232_244	GAKLRKVSKQEEA	[239]	[]	1000	P50552	Vasodilator-stimulated phosphoprotein (VASP).
12,10	GBRB2_427_439	SRLRRRASQLKIT	[427, 434]	[439]	200	P47870	Gamma-aminobutyric acid receptor subunit beta-2 precursor (GABA(A)receptor subunit beta-2).
12,11	PYGL_8_20	QEKRRQISIRGIV	[15]	[]	200	P06737	Glycogen phosphorylase, liver form (EC 2.4.1.1).
12,12	TY3H_65_77	FIGRRQSLIEDAR	[71]	[]	200	P07101	Tyrosine 3-monooxygenase (EC 1.14.16.2) (Tyrosine 3-hydroxylase) (TH).
