# Supplementary material for: Loss of carnitine palmitoyltransferase 1a reduces docosahexaenoic acid-containing phospholipids and drives sexually dimorphic liver disease in mice
Source: Mol Metab. 2023 Oct 4;78:101815. doi: 10.1016/j.molmet.2023.101815 (PMC10568566; doi:10.1016/j.molmet.2023.101815)
Supplement: Multimedia component 2 [file mmc2.pdf]

Supplemental Table 2. STK Scores and Rankings Male LKO versus Control.

Chip	Kinase Uniprot ID	Kinase Name	UKA Mean Final Score	KRSA Z-Score
STK	P51817	PRKX	3.111809995	2.529633673
STK	Q13976	PKG1	3.158651268	2.445281341
STK	P17612	PKA[alpha]	3.443324027	2.356425466
STK	Q86V86	Pim3	1.308285831	2.223978899
STK	Q16539	MAPK14	0.802383313	-2.156149343
STK	Q13237	PKG2	3.512189444	2.135018211
STK	Q16644	MAPKAPK3	1.204716045	1.925710638
STK	P11802	CDK4	0.556943338	-1.847471793
STK	P49137	MAPKAPK2	1.256764996	1.772399097
STK	Q9Y6S9	RSKL2	1.342948469	1.723372384
STK	P45983	JNK1	0.306887231	-1.717927171
STK	P45984	JNK2	0.363192669	-1.64946
STK	P53779	JNK3	0.354832933	-1.637249591
STK	P11309	Pim1	1.080952117	1.601519942
STK	Q9UBS0	p70S6K[beta]	3.192738193	1.575058299
STK	Q00535	CDK5	0.729472959	-1.525831101
STK	P24941	CDK2	0.309493576	-1.388225822
STK	P49840	GSK3[alpha]	0.159335565	-1.276965997
STK	Q96L96	AlphaK1	0.920202592	1.24626505
STK	O75676	MSK2	0.113929424	1.244412482
STK	Q00526	CDK3	0.961761287	-1.239957819
STK	Q92772	CDKL2	1.574792073	1.232795042
STK	P42345	mTOR/FRAP	0.394560245	-1.184449408
STK	Q00534	CDK6	0.539734886	-1.163128249
STK	P06493	CDC2/CDK1	1.005935185	-1.144363283
STK	P68400	CK2[alpha]1	1.353155423	-1.142067594
STK	Q9HBY8	SGK2	1.492588211	1.127073508
STK	P41279	COT	2.193056892	-1.111654847
STK	Q15759	p38[beta]	0.473979192	-1.034682702
STK	O75582	MSK1	0.279723604	1.003195413
STK	Q13464	ROCK1	0.835902842	0.956265635
STK	P53778	p38[gamma]	0.265015714	-0.954828781
STK	O14965	AurA/Aur2	0.015671136	-0.946553347
STK	P28482	ERK2	0.321431267	-0.940762732
STK	Q00532	CDKL1	0.953171277	-0.936833094
STK	Q9P1W9	Pim2	1.654101631	0.918937472
STK	Q96Q40	PFTAIRE2	1.275658344	-0.871056317
STK	O60285	NuaK1	1.347129647	-0.870020344
STK	P50613	CDK7	0.297692008	0.858574411
STK	P48730	CK1[delta]	3.308379687	0.844231234
STK	Q05513	PKC[zeta]	0.217103998	-0.8273527
STK	Q15139	PKD1	2.288134594	0.818032911

STK	Q9BWU1	CDK11	0.395290293	-0.810860528
STK	Q05655	PKC[delta]	1.648729418	0.802004893
STK	Q9UIK4	DAPK2	1.452185287	-0.801664316
STK	P17252	PKC[alpha]	1.440882864	0.784405507
STK	O14757	CHK1	0.812534559	0.783460579
STK	P27361	ERK1	0.355786452	-0.767228048
STK	Q9UQM7	CaMK2[alpha]	2.06321783	0.727911225
STK	Q13131	AMPK[alpha]1	1.559729428	0.712480458
STK	Q14164	IKK[epsilon]	0.517511478	0.707545496
STK	O76039	CDKL5	0.924661328	0.693455456
STK	O94921	PFTAIRE1	1.576398879	-0.686932972
STK	P31751	Akt2/PKB[beta]	2.23528387	0.684157997
STK	Q13627	DYRK1A	0.878424816	0.682118833
STK	P23443	p70S6K	1.259665334	0.657977832
STK	O43293	DAPK3	1.154695654	-0.651974963
STK	Q13535	ATR	1.532426525	-0.645059086
STK	Q02156	PKC[epsilon]	1.151991027	0.641736374
STK	Q13164	ERK5	0.857342792	-0.541072671
STK	P15056	BRAF	1.687822644	-0.520283125
STK	P49674	CK1[epsilon]	2.331926044	0.514907145
STK	Q15418	RSK3	1.312410551	-0.50241818
STK	P04049	RAF1	0.773789417	-0.502183724
STK	Q8NI60	ADCK3	0.298337511	-0.497094973
STK	P49841	GSK3[beta]	0.159837603	-0.485460908
STK	O96017	CHK2	1.523917342	0.466538477
STK	P05771	PKC[beta]	0.252050689	-0.463127953
STK	O14920	IKK[beta]	1.669783497	0.428529467
STK	P51812	RSK2	1.006270391	0.417001964
STK	Q16512	PKN1/PRK1	0.521640261	0.406786354
STK	O75116	ROCK2	1.020509203	0.387205506
STK	P05129	PKC[gamma]	0.708389234	-0.385589925
STK	P24723	PKC[eta]	0.702617592	0.373518155
STK	O15264	p38[delta]	0.173671854	-0.354647515
STK	Q15131	CDK10	1.261302099	0.346928941
STK	P31749	Akt1/PKB[alpha]	2.740098368	0.292863781
STK	Q00537	PCTAIRE2	0.883977021	0.287881876
STK	Q8IWB6	SgK307	1.178288967	-0.280337815
STK	Q8TD08	ERK7	0.814559844	0.262998604
STK	O43930	PRKY	1.710450348	0.23740726
STK	P48729	CK1[alpha]	2.05883531	-0.22299891
STK	O15111	IKK[alpha]	3.117044722	0.20052033
STK	P50750	CDK9	0.164390034	0.132980272
STK	Q16566	CaMK4	2.593234543	0.108770163

STK	Q9UHD2	TBK1	1.042579242	-0.08094341
STK	Q04759	PKC[theta]	0.918340295	-0.07287373
STK	P16066	ANP[alpha]	1.365475179	0.052870348
STK	Q15349	RSK1/p90RSK	1.62507002	-0.049420269
STK	Q13153	PAK1	0.080711017	-0.032328312
STK	Q96GD4	AurB/Aur1	0.116466043	-0.023903083
STK	P41743	PKC[iota]	1.121345481	0.022648981
STK	P53355	DAPK1	0.598446019	0.001821918
