# Supplementary material for: Loss of carnitine palmitoyltransferase 1a reduces docosahexaenoic acid-containing phospholipids and drives sexually dimorphic liver disease in mice
Source: Mol Metab. 2023 Oct 4;78:101815. doi: 10.1016/j.molmet.2023.101815 (PMC10568566; doi:10.1016/j.molmet.2023.101815)
Supplement: Multimedia component 3 [file mmc3.pdf]

Supplemental Table 3. STK Scores and Rankings Female LKO versus Control.

Chip	Kinase Uniprot ID	Kinase Name	UKA Mean Final Score	KRSA Z-Score
STK	Q9UBS0	p70S6K[beta]	1.017341984	2.933655215
STK	P51817	PRKX	1.942685529	2.666579126
STK	P27361	ERK1	0.399523427	-2.567561766
STK	Q9UHD2	TBK1	0.363808154	-2.51196494
STK	P28482	ERK2	0.42285169	-2.457333075
STK	Q16539	MAPK14	0.634491863	-2.431932472
STK	P17612	PKA[alpha]	3.194708056	2.217966944
STK	P45983	JNK1	0.717784731	-2.03310928
STK	P31749	Akt1/PKB[alpha]	2.241460632	1.933808204
STK	P53779	JNK3	0.71979217	-1.888316325
STK	P06493	CDC2/CDK1	0.592942691	-1.885753009
STK	O15264	p38[delta]	0.574215613	-1.883733192
STK	Q13976	PKG1	1.761794596	1.861818926
STK	Q9Y6S9	RSKL2	1.110588827	1.837524663
STK	P24941	CDK2	0.628888811	-1.773208064
STK	P41743	PKC[iota]	0.562723253	-1.762719071
STK	Q16644	MAPKAPK3	0.969017277	1.686049842
STK	Q86V86	Pim3	1.052172601	1.667473768
STK	Q96L96	AlphaK1	8.70E-04	-1.626209413
STK	P45984	JNK2	0.799735289	-1.620462457
STK	Q14164	IKK[epsilon]	1.173806428	-1.604896154
STK	Q9UIK4	DAPK2	0.979700427	-1.481603616
STK	Q15759	p38[beta]	0.472812624	-1.466695976
STK	O96017	CHK2	1.354050318	1.449775095
STK	Q8NI60	ADCK3	0.86262824	1.41117678
STK	P11309	Pim1	1.141398696	1.392256907
STK	P49840	GSK3[alpha]	1.83955768	-1.347157303
STK	P53778	p38[gamma]	0.476992767	-1.291731828
STK	Q13464	ROCK1	0.456646091	-1.285532889
STK	O94921	PFTAIRE1	1.614393972	1.262696415
STK	O75676	MSK2	1.754244576	1.254151907
STK	P15056	BRAF	1.516327136	-1.249582085
STK	Q9HBY8	SGK2	1.67746092	1.208933976
STK	P51812	RSK2	2.088153928	1.145524947
STK	P48729	CK1[alpha]	0.72670781	-1.129774788
STK	P16066	ANP[alpha]	0.30772192	-1.129290353
STK	P49137	MAPKAPK2	1.353509862	1.125169053
STK	P49674	CK1[epsilon]	0.34452163	-1.1196131
STK	P31751	Akt2/PKB[beta]	2.298553684	1.098341817
STK	P41279	COT	1.712197337	-1.093630141
STK	P49841	GSK3[beta]	1.845537345	-1.088881206
STK	Q00534	CDK6	0.837452808	-1.08457661
STK	O75582	MSK1	1.750172301	1.018943256
STK	O14965	AurA/Aur2	0.6957088	0.965605408
STK	Q00532	CDKL1	0.580687662	-0.924763198
STK	P48730	CK1[delta]	0.469754592	-0.922241172
STK	Q8IWB6	SgK307	0.207710433	-0.909748426
STK	Q16566	CaMK4	1.027563715	-0.908173196

STK	O75116	ROCK2	0.966224207	-0.900332679
STK	Q00535	CDK5	0.852275386	-0.892377812
STK	P50613	CDK7	0.885639837	-0.883741579
STK	O60285	NuaK1	1.155036027	-0.883713334
STK	Q13237	PKG2	1.651737962	0.8763904
STK	Q13153	PAK1	0.484710401	-0.861035142
STK	Q04759	PKC[theta]	2.022212117	0.860735607
STK	Q13164	ERK5	0.429144742	-0.84791894
STK	P23443	p70S6K	1.066225223	0.812421518
STK	O43930	PRKY	0.633005657	0.759369219
STK	Q9BWU1	CDK11	0.947924679	-0.727907435
STK	Q00526	CDK3	1.323591928	-0.723564057
STK	Q9P1W9	Pim2	1.039275033	0.715581707
STK	Q9UQM7	CaMK2[alpha]	2.028239866	0.705704408
STK	Q13627	DYRK1A	0.784072915	0.705088328
STK	Q8TD08	ERK7	0.263284866	-0.696495476
STK	O43293	DAPK3	0.58743602	-0.651338556
STK	Q00537	PCTAIRE2	0.408775068	-0.60623194
STK	O15111	IKK[alpha]	1.229498358	-0.511868562
STK	P17252	PKC[alpha]	1.953322049	0.493057703
STK	P42345	mTOR/FRAP	0.395380641	-0.462426092
STK	P05771	PKC[beta]	0.679332593	-0.459115533
STK	O14920	IKK[beta]	1.935033939	0.458253793
STK	P24723	PKC[eta]	1.097582503	0.452689499
STK	P04049	RAF1	0.811003937	-0.445676118
STK	Q15131	CDK10	0.869295638	0.421894836
STK	Q05655	PKC[delta]	1.539266638	0.416866647
STK	O14757	CHK1	0.501981083	-0.400881993
STK	Q92772	CDKL2	0.867130045	0.286952491
STK	P68400	CK2[alpha]1	1.658146824	-0.284493007
STK	Q16512	PKN1/PRK1	2.586247777	-0.241828628
STK	Q15418	RSK3	1.650810144	0.2382695
STK	Q96Q40	PFTAIRE2	0.837052384	-0.219568702
STK	O76039	CDKL5	0.401753149	0.203647213
STK	P50750	CDK9	1.380166415	0.181682903
STK	Q05513	PKC[zeta]	0.502175686	-0.178486987
STK	Q02156	PKC[epsilon]	1.269600342	-0.175910746
STK	Q13131	AMPK[alpha]1	2.064292842	0.175882774
STK	P05129	PKC[gamma]	1.122933401	0.150794753
STK	P11802	CDK4	0.82155846	-0.045681402
STK	Q15139	PKD1	1.55465892	0.029114726
STK	Q15349	RSK1/p90RSK	1.965969489	0.016764465
STK	Q96GD4	AurB/Aur1	0.721626555	0.011984258
STK	P53355	DAPK1	1.683487177	0.011740595
STK	Q13535	ATR	0.446142034	-0.004727717
