# Supplementary material for: Loss of carnitine palmitoyltransferase 1a reduces docosahexaenoic acid-containing phospholipids and drives sexually dimorphic liver disease in mice
Source: Mol Metab. 2023 Oct 4;78:101815. doi: 10.1016/j.molmet.2023.101815 (PMC10568566; doi:10.1016/j.molmet.2023.101815)
Supplement: Multimedia component 4 [file mmc4.pdf]

Supplemental Table 4. Mouse primers used for real-time PCR.

**Gene**	**Sequence (5' – 3')**	**Forward/Reverse**
Cpt1a	AGTGGCCTCACAGACTCCAG	Forward
	GCCCATGTTGTACAGCTTCC	Reverse
Cpt1b	GCTGCTTGCACATTTGTGTT	Forward
	TGAGTGACTGGTGGGAAGAA	Reverse
Pparγ1	AACAAGACTACCCTTTACTGAAATTACCA	Forward
	CACAGAGCTGATTCCGAAGTTG	Reverse
Pparγ2	CCAGAGCATGGTGCCTTCGCT	Forward
	CAGCAACCATTGGGTCAG	Reverse
18S	GTAACCCGTTGAACCCCATT	Forward
	CCATCCAATCGGTAGTAGCG	Reverse
Hprt	CACGCAACCAGGAAGTAGAA	Forward
	AGAGCGAGAACGAACAGATTA	Reverse
