# Supplementary material for: Loss of carnitine palmitoyltransferase 1a reduces docosahexaenoic acid-containing phospholipids and drives sexually dimorphic liver disease in mice
Source: Mol Metab. 2023 Oct 4;78:101815. doi: 10.1016/j.molmet.2023.101815 (PMC10568566; doi:10.1016/j.molmet.2023.101815)
Supplement: Multimedia component 5 [file mmc5.pdf]

Supplemental Table 5. Antibodies used for immunoblotting.

**Protein**	**Catalog Information**	**Species & Clonality**	**Dilution**
CPT1a	128568	Mouse Monoclonal	1:1000
CPT1b	22170-1-AP	Rabbit Polyclonal	1:1000
Vinculin	NB600-1293	Mouse Monoclonal	1:1000
PEMT	Gift from Dr. René Jacobs (rabbit)		1:1000
PLIN2	NB110-40877	Rabbit Polyclonal	1:1000
PLIN5	GP31	Pig Polyclonal	1:1000
VDAC	CS-4866S	Rabbit Polyclonal	1:1000
GAPDH	5174T	Rabbit Monoclonal	1:1000
ATGL	2138	Rabbit Polyclonal	1:1000
CGI-58	NB110-41576	Rabbit Polyclonal	1:1000
G0S2	Gift from Dr. Jun Liu (rabbit)		1:1000
HSL	4107	Rabbit Polyclonal	1:1000
MGL	sc-398942	Mouse Monoclonal	1:1000
PKA Substrate	9624	Mouse Monoclonal	1:1000
