# Supplementary material for: Loss of carnitine palmitoyltransferase 1a reduces docosahexaenoic acid-containing phospholipids and drives sexually dimorphic liver disease in mice
Source: Mol Metab. 2023 Oct 4;78:101815. doi: 10.1016/j.molmet.2023.101815 (PMC10568566; doi:10.1016/j.molmet.2023.101815)
Supplement: Multimedia component 6 — Supplemental Figure 1. CPT1a Deletion in the Liver Reduces Fasting Ketones In Response to LFD-Feeding. (A) An overview of the experimental design. (B-D) Liver CPT1a RNA and protein levels were measured by qPCR (B) and western blot (C) followed by densitometry (D), respectively (n = 5). Vinculin is used as a loading control for immunoblotting. (E) RNA levels of Cpt1b were measured in livers of LFD-fed control and LKO mice by qPCR (n = 8). For all qPCR analyses, housekeeping genes Tbp and Hprt were averaged and used for normalization. (F) β-hydroxybutyrate levels were measured from plasma of fasted mice fed LFD (n = 7–8). Significance was determined by two-way ANOVA with Tukey’s multiple comparison post hoc analysis. ∗P < 0.05; ∗∗P < 0.01; ∗∗∗P < 0.001; ∗∗∗∗P < 0.0001. Supplemental Figure 2. CPT1b RNA and Protein Levels Are Low in Liver. (A) Total read counts for Cpt1a and Cpt1b in FPKM (fragments per kilobase million) from bulk RNA-sequencing data on HFD-fed control male livers (n = 6). (B) Total read counts for Cpt1b in male control and Cpt1a LKO mice fed a HFD (n = 6). (C) A comparison of CPT1b protein expression from liver and brown adipose tissue (BAT) of female control mice fed HFD. Vinculin was used as a loading control. Significance was determined by an unpaired Student’s t-test. ∗P < 0.05; ∗∗∗∗P < 0.0001. Supplemental Figure 3. Liver-specific CPT1a Deletion Has No Effect on Body Weight at Baseline and Response to LFD-Feeding. (A-C) Baseline body weights (A), percent (%) fat (B) and lean (C) mass in control and LKO mice at 6–8 weeks of age (n = 15–20). (D, E) Percent body weight (D) and fat (E) mass throughout the study in response to LFD-feeding (n = 8–12). Significance was determined by two-way ANOVA with Tukey’s multiple comparison post hoc analysis. ∗∗∗∗P < 0.0001. Supplemental Figure 4. LFD-Fed Control and LKO Mice Exhibit Similar Glucose Tolerance. After 10- and 12-weeks of LFD-feeding, male and female control and LKO mice underwent intraperitoneal gluco [file mmc6.pptx]

## Slide 1
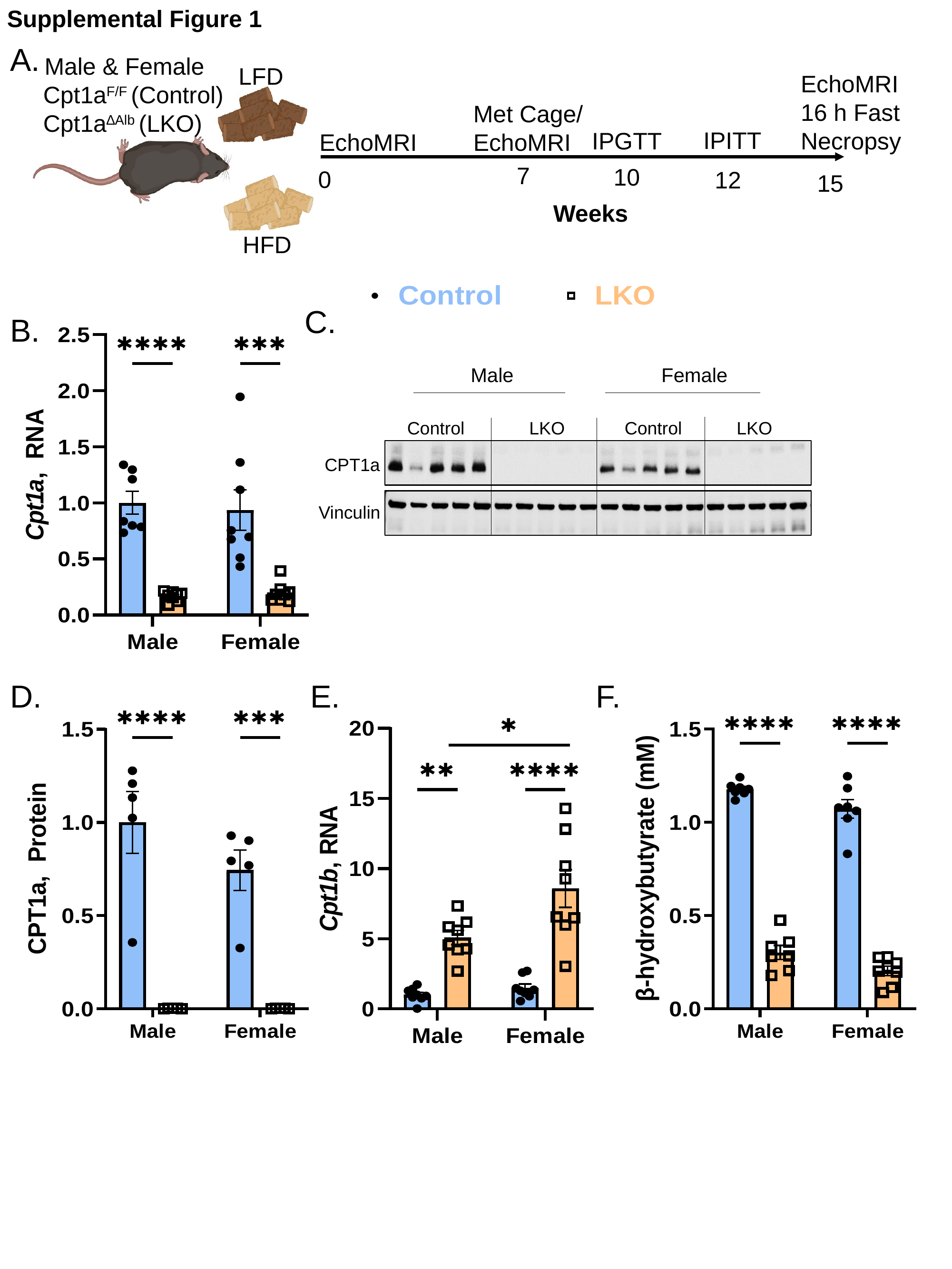

Supplemental Figure 1
A.
Male & Female
LFD
EchoMRI
16 h Fast
Necropsy
Cpt1aF/F (Control)
Cpt1a∆Alb (LKO)
IPITT
IPGTT
EchoMRI
10
0
12
15
Weeks
HFD
Met Cage/
EchoMRI
7
C.
B.
 Male Female
 Control LKO Control LKO
CPT1a
Vinculin
D.
E.
F.

## Slide 2
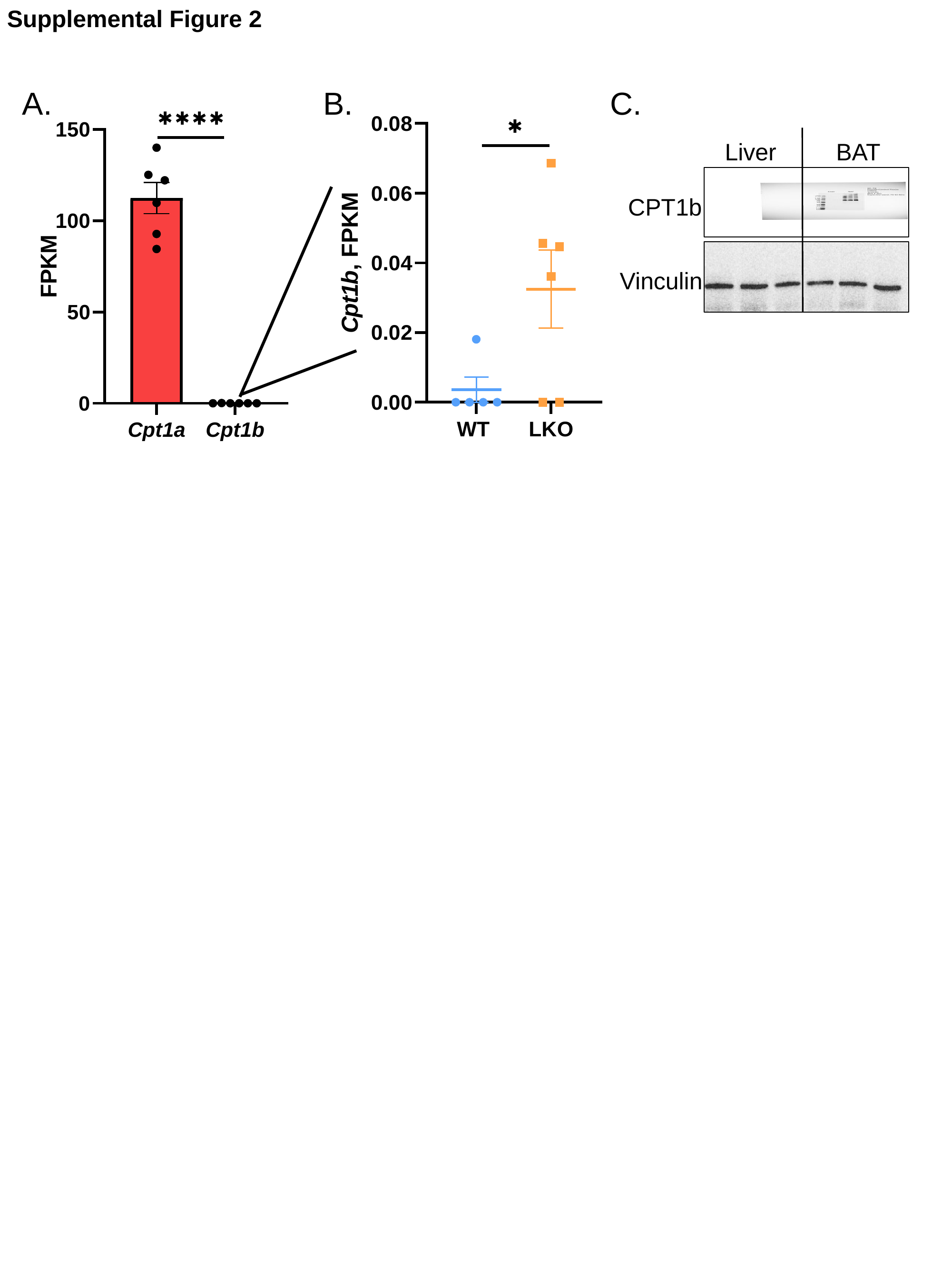

Supplemental Figure 2
C.
B.
A.
 Liver BAT
CPT1b
Vinculin

## Slide 3
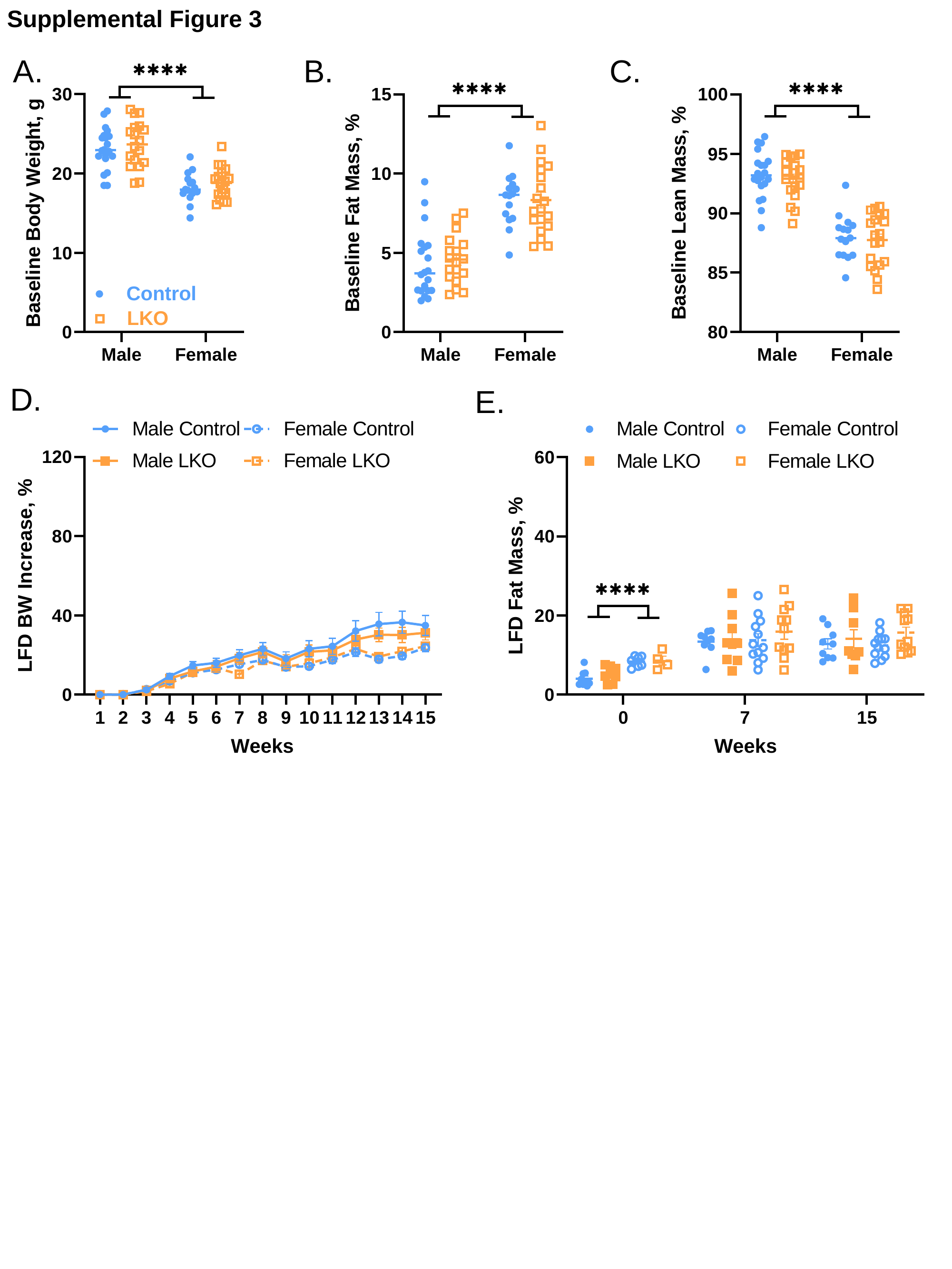

Supplemental Figure 3
A.
B.
C.
D.
E.

## Slide 4
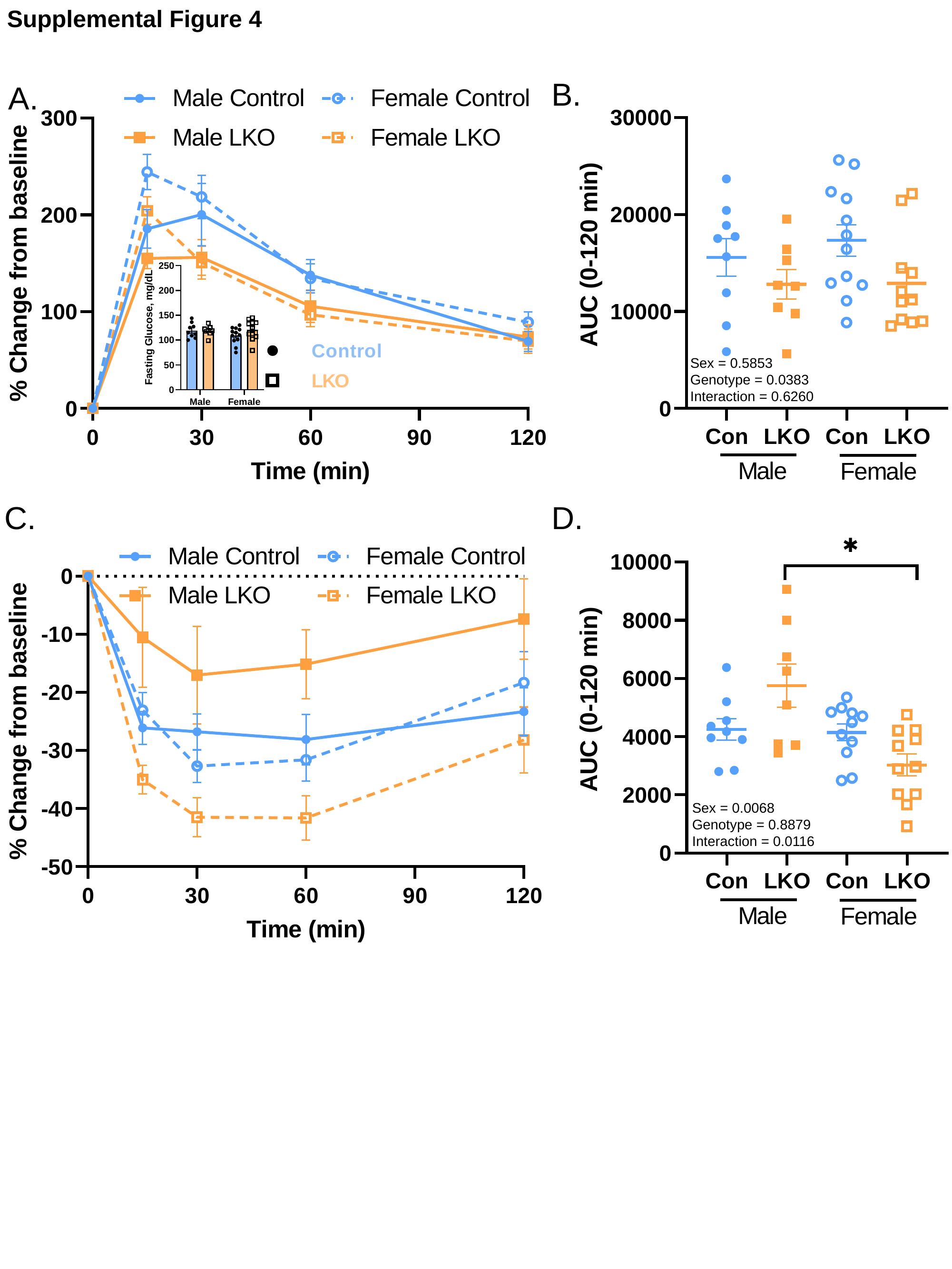

Supplemental Figure 4
B.
A.
Sex = 0.5853
Genotype = 0.0383
Interaction = 0.6260
D.
C.
Sex = 0.0068
Genotype = 0.8879
Interaction = 0.0116

## Slide 5
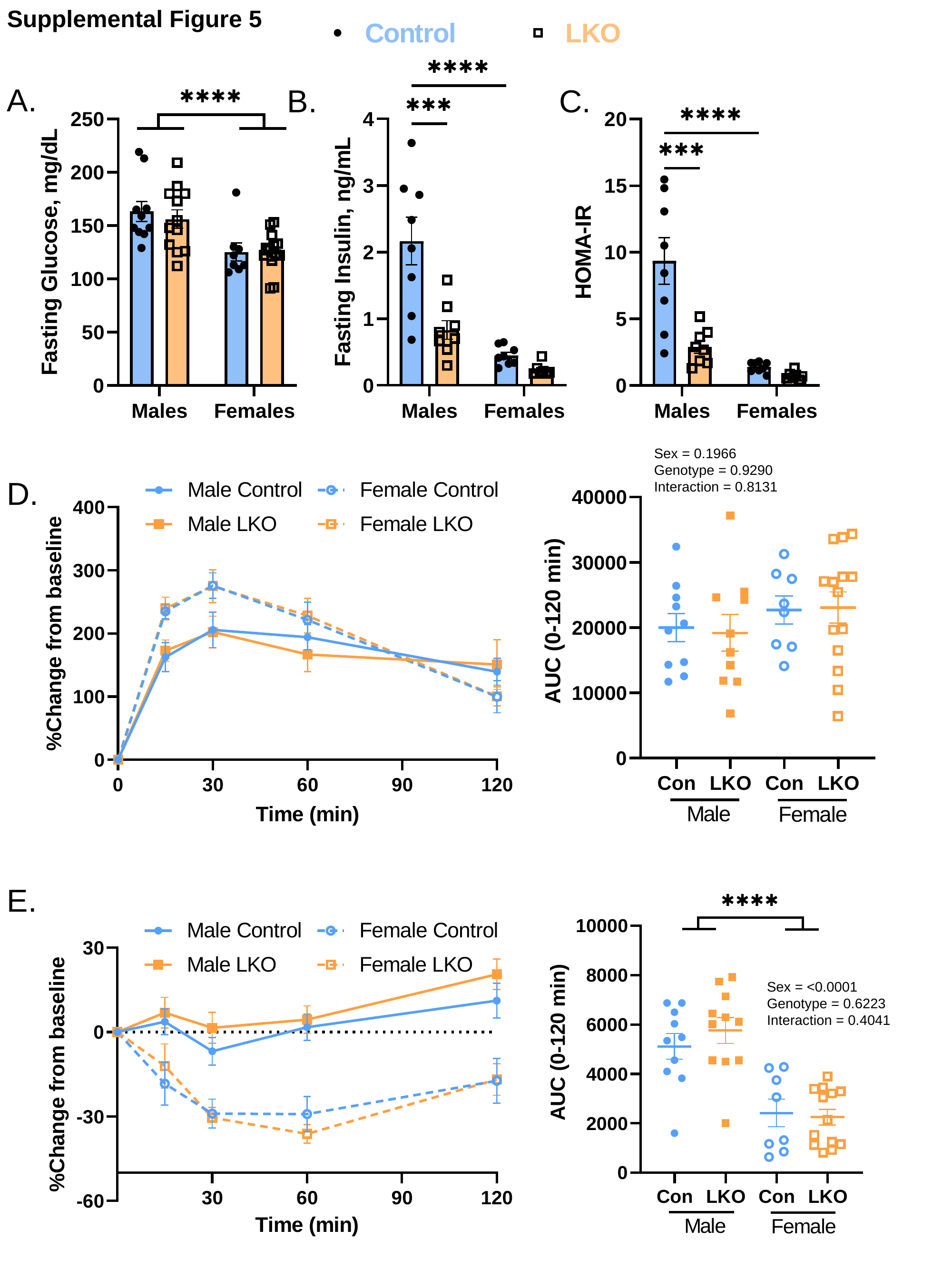

Supplemental Figure 5
A.
B.
C.
D.
E.
Sex = 0.1966
Genotype = 0.9290
Interaction = 0.8131
Sex = <0.0001
Genotype = 0.6223
Interaction = 0.4041

## Slide 6
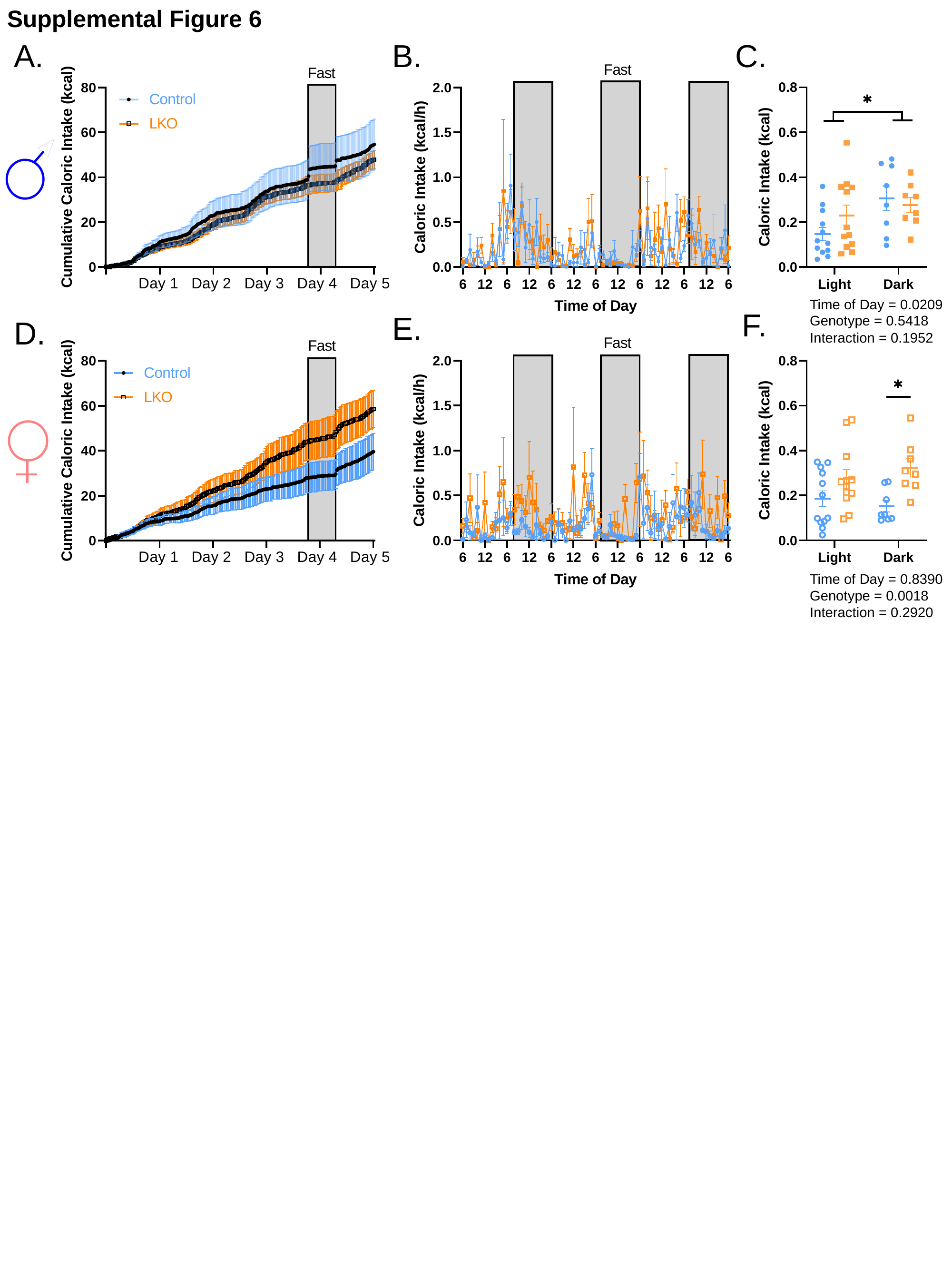

Supplemental Figure 6
A.
C.
B.
Time of Day = 0.0209
Genotype = 0.5418
Interaction = 0.1952
F.
E.
D.
Time of Day = 0.8390
Genotype = 0.0018
Interaction = 0.2920

## Slide 7
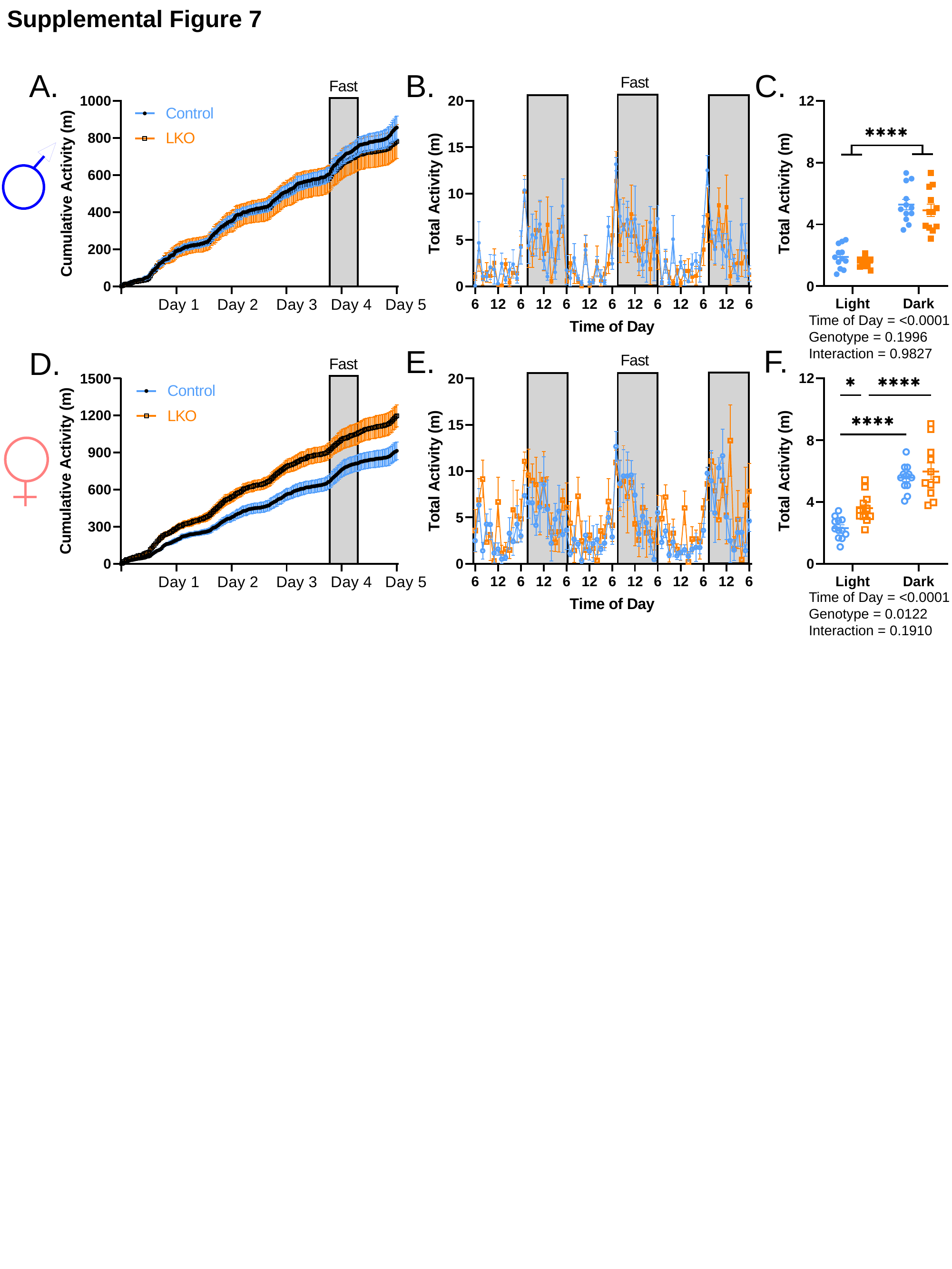

Supplemental Figure 7
C.
B.
A.
Time of Day = <0.0001
Genotype = 0.1996
Interaction = 0.9827
F.
E.
D.
Time of Day = <0.0001
Genotype = 0.0122
Interaction = 0.1910

## Slide 8
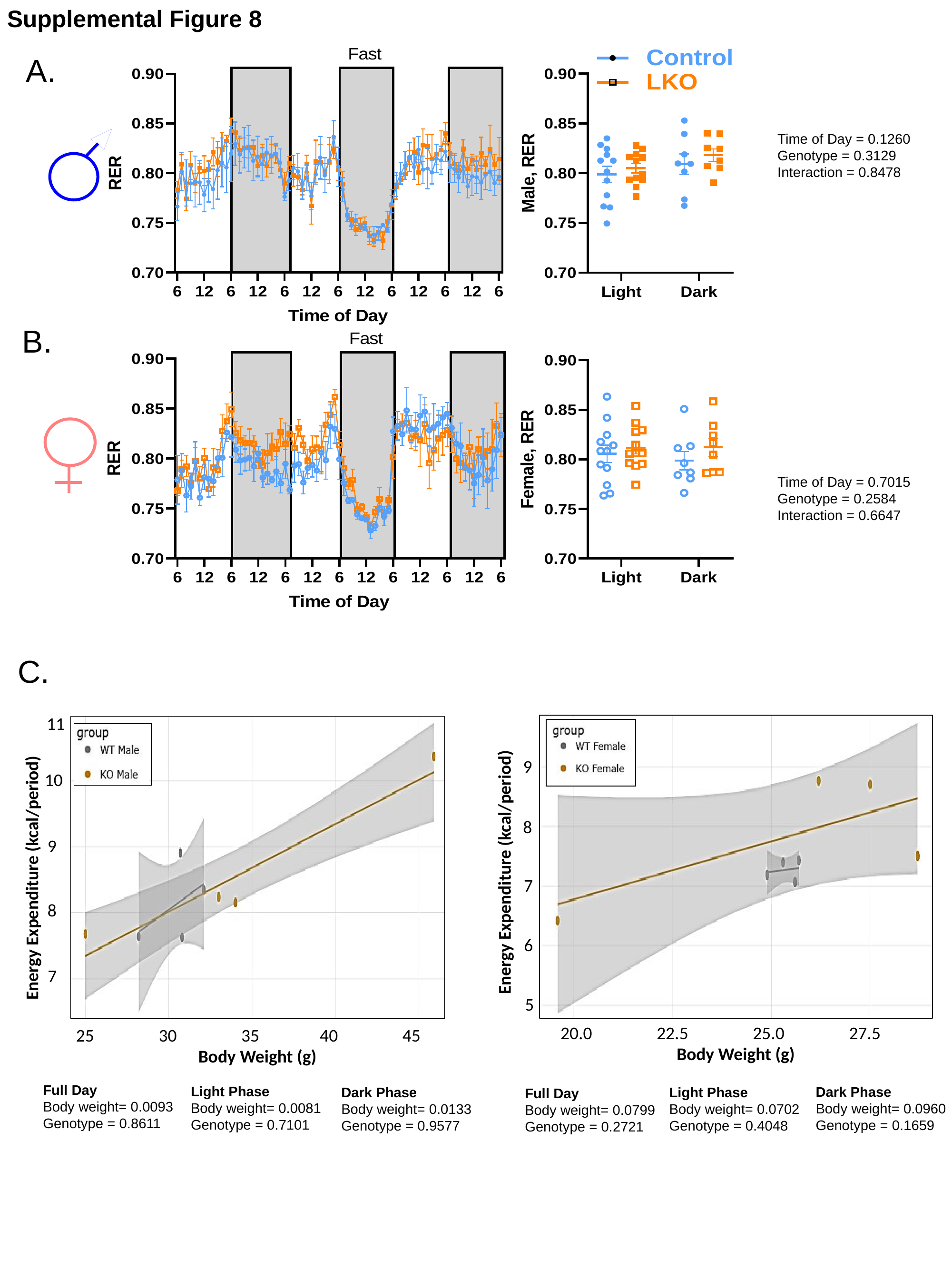

Supplemental Figure 8
A.
Time of Day = 0.1260
Genotype = 0.3129
Interaction = 0.8478
B.
Time of Day = 0.7015
Genotype = 0.2584
Interaction = 0.6647
C.
9
8
Energy Expenditure (kcal/period)
7
5
 20.0 22.5 25.0 27.5
Body Weight (g)
6
10
9
Energy Expenditure (kcal/period)
8
 25 30 35 40 45
Body Weight (g)
7
11
Full Day
Body weight= 0.0093
Genotype = 0.8611
Light Phase
Body weight= 0.0081
Genotype = 0.7101
Dark Phase
Body weight= 0.0960
Genotype = 0.1659
Light Phase
Body weight= 0.0702
Genotype = 0.4048
Dark Phase
Body weight= 0.0133
Genotype = 0.9577
Full Day
Body weight= 0.0799
Genotype = 0.2721

## Slide 9
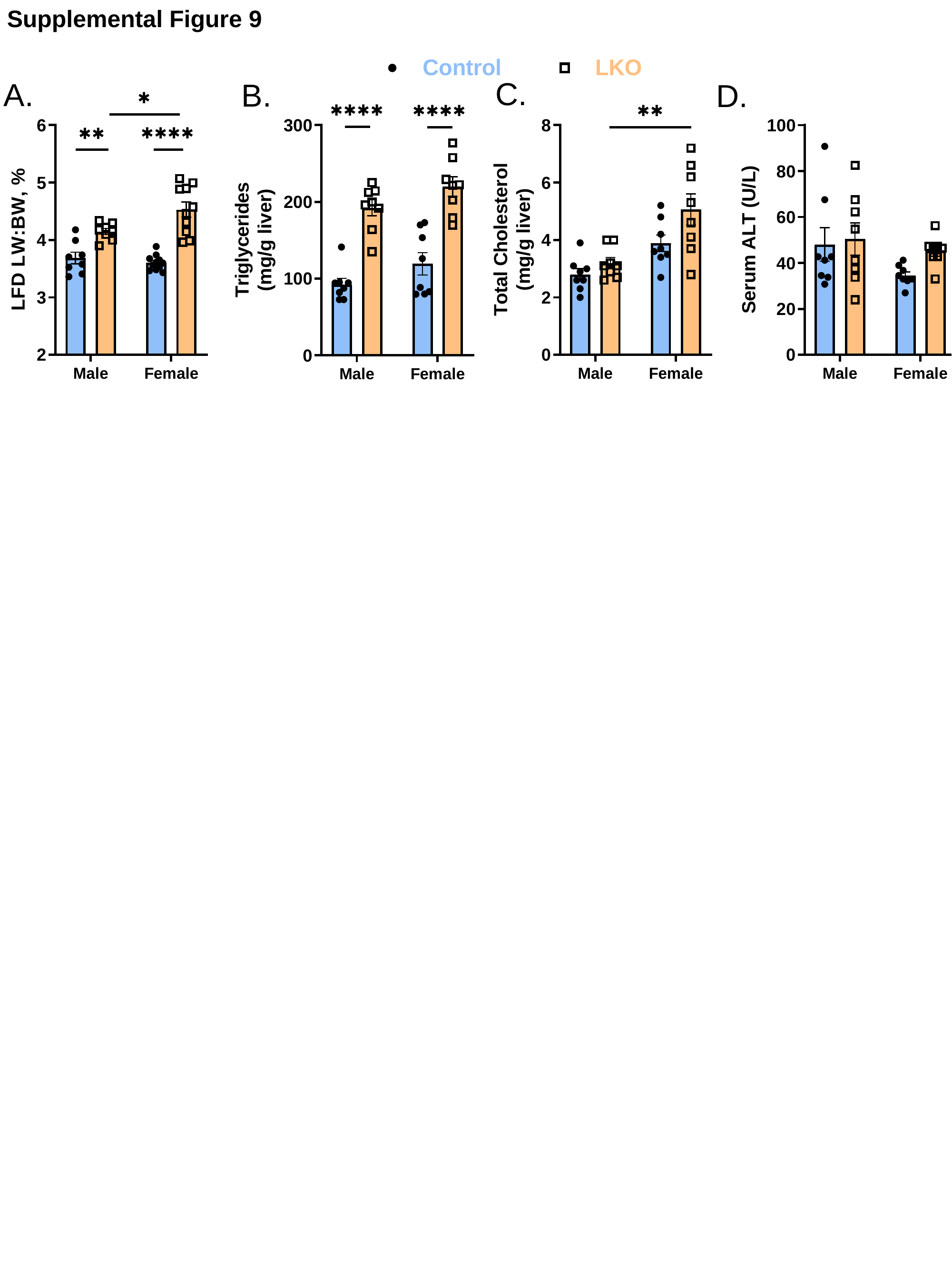

Supplemental Figure 9
C.
A.
B.
D.

## Slide 10
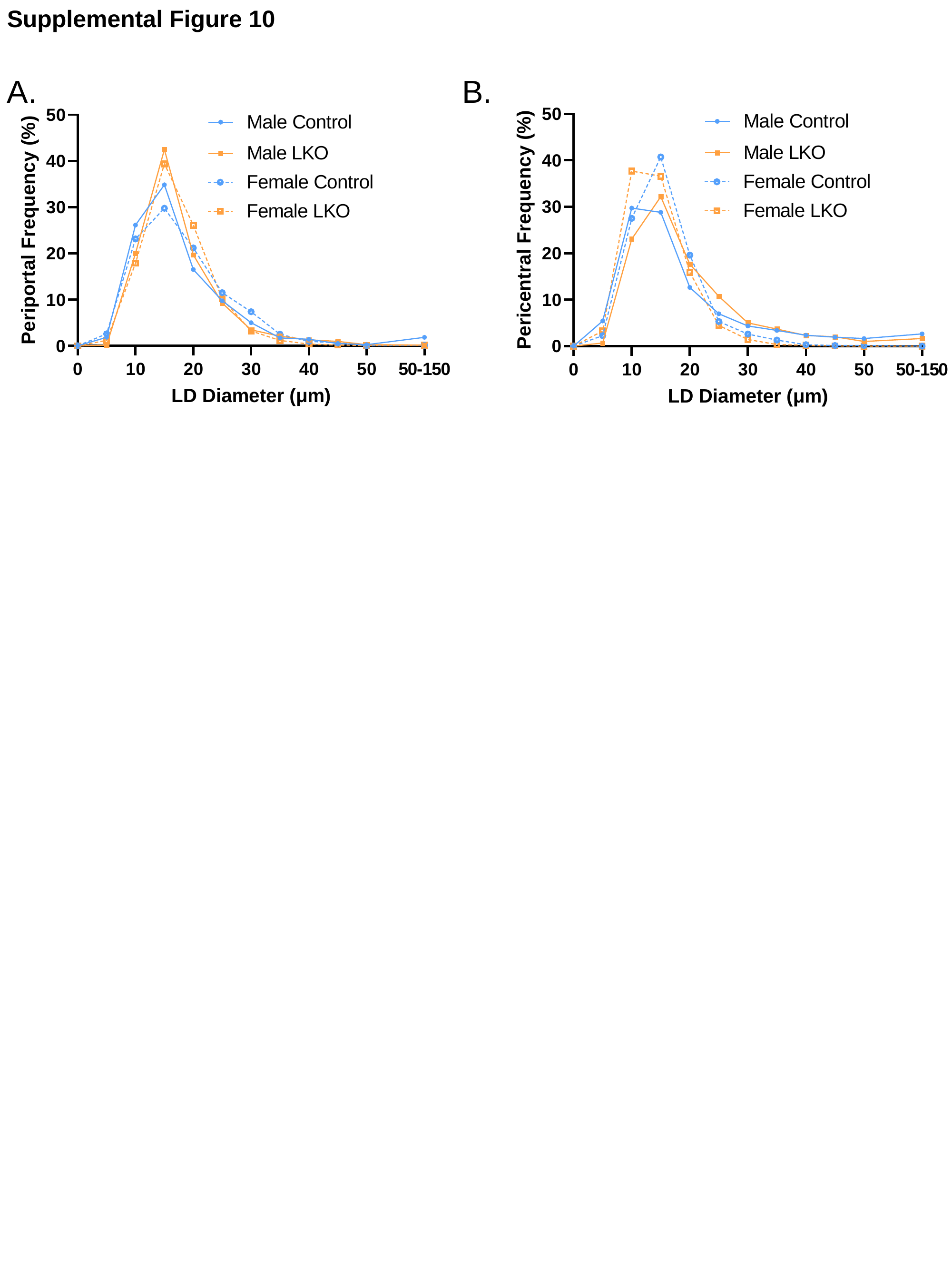

Supplemental Figure 10
A.
B.

## Slide 11
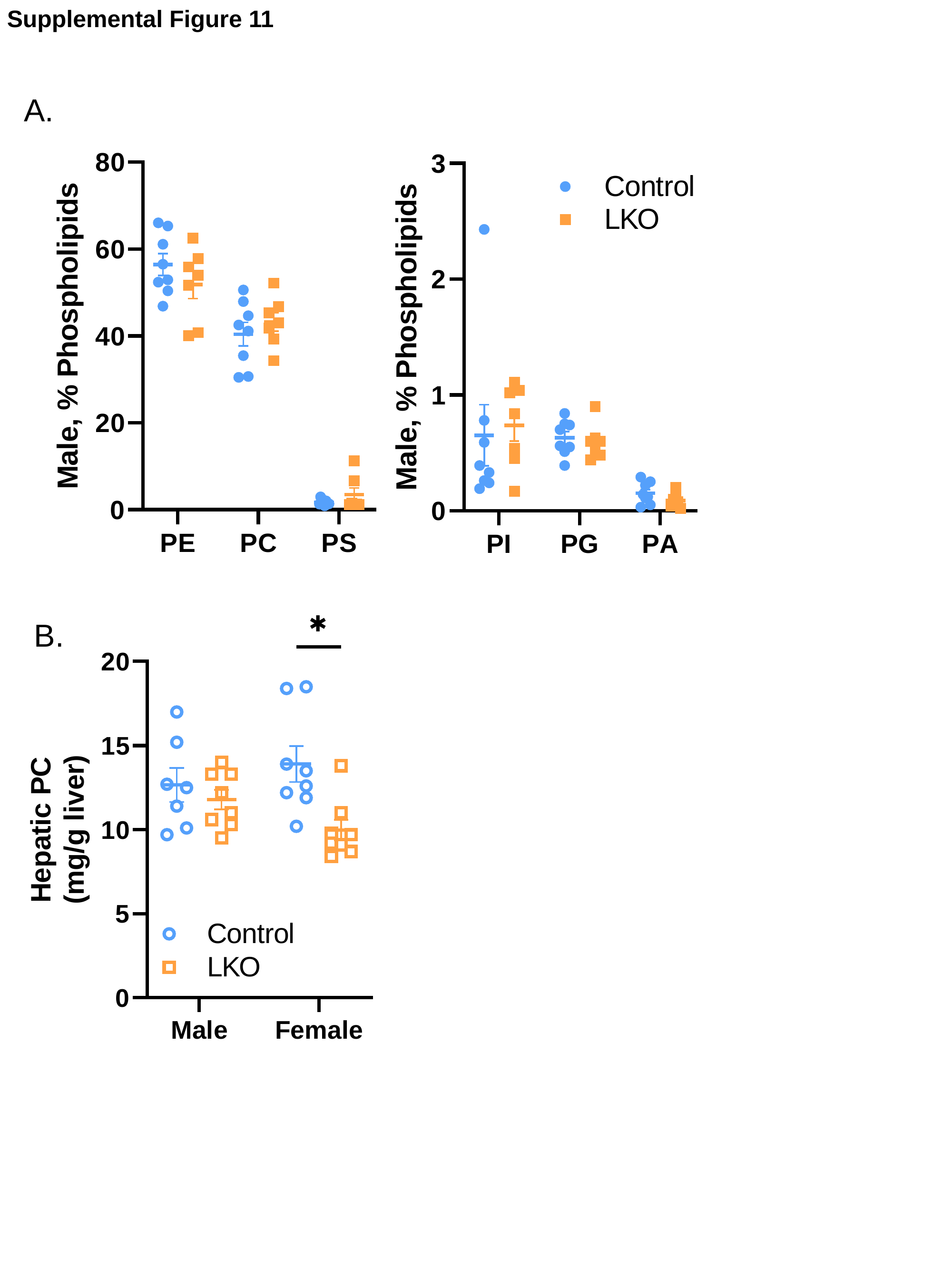

Supplemental Figure 11
A.
B.

## Slide 12
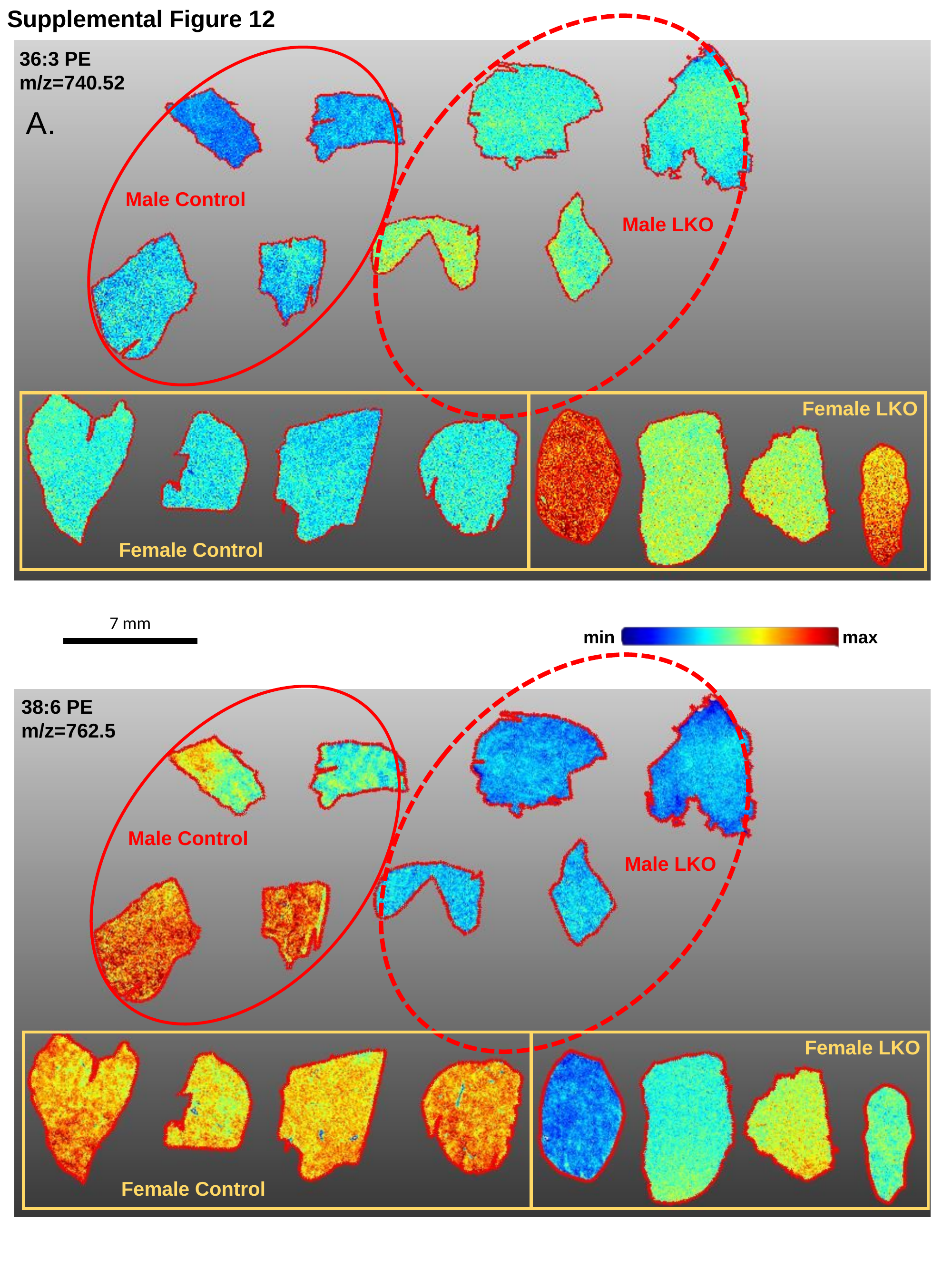

Supplemental Figure 12
36:3 PE
m/z=740.52
A.
Male Control
Male LKO
Female LKO
Female Control
7 mm
min
max
38:6 PE
m/z=762.5
Male Control
Male LKO
Female LKO
Female Control

## Slide 13
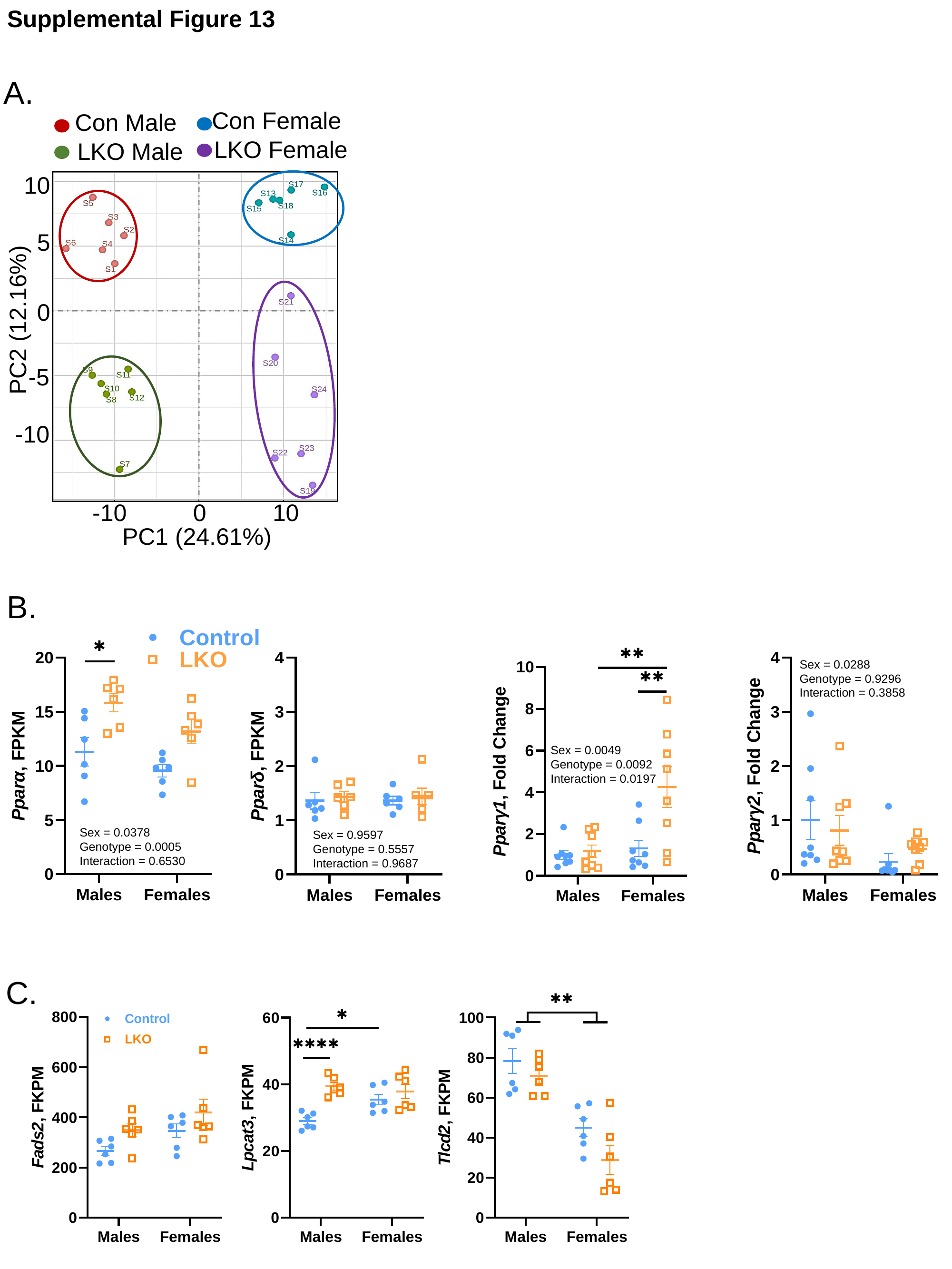

Supplemental Figure 13
A.
Con Female
Con Male
LKO Male
10
5
PC2 (12.16%)
0
-5
-10
 -10 0 10
PC1 (24.61%)
LKO Female
B.
Sex = 0.0288
Genotype = 0.9296
Interaction = 0.3858
Sex = 0.0049
Genotype = 0.0092
Interaction = 0.0197
Sex = 0.0378
Genotype = 0.0005
Interaction = 0.6530
Sex = 0.9597
Genotype = 0.5557
Interaction = 0.9687
C.

## Slide 14
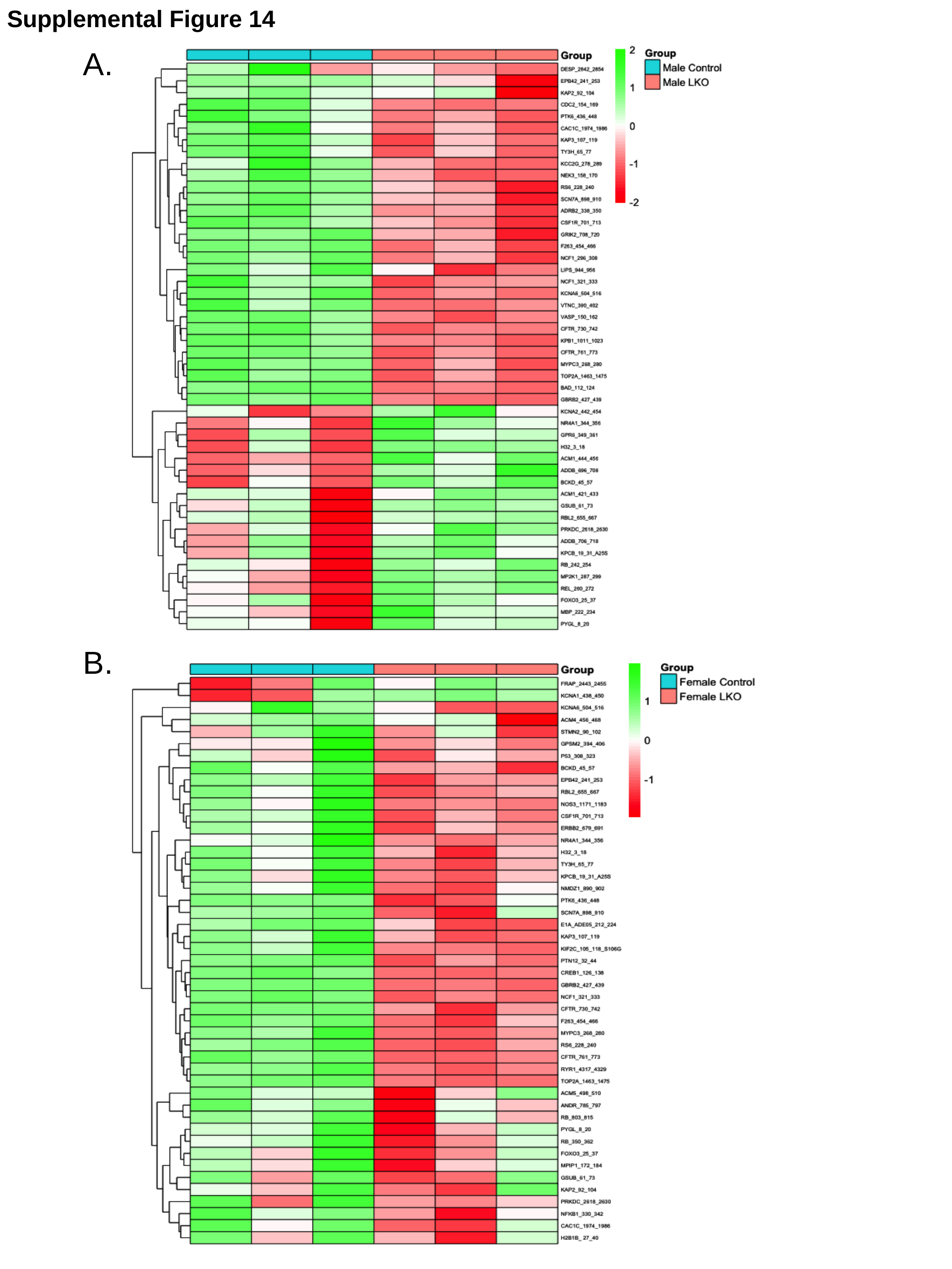

Supplemental Figure 14
A.
B.

## Slide 15
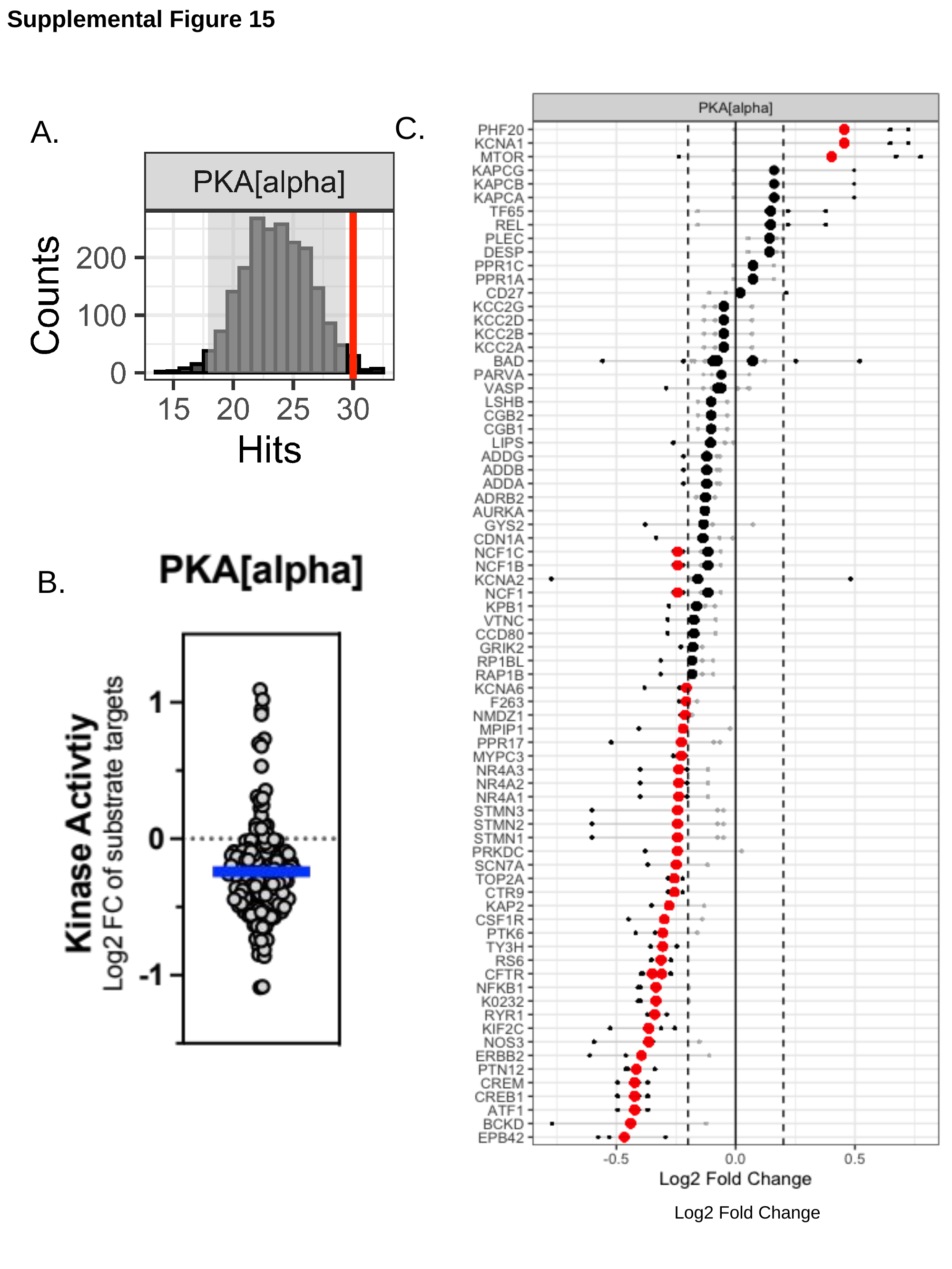

Supplemental Figure 15
C.
A.
B.
Log2 Fold Change
